# Supplementary material for: Minimal lactazole scaffold for in vitro thiopeptide bioengineering
Source: Nat Commun. 2020 May 8;11:2272. doi: 10.1038/s41467-020-16145-4 (PMC7210931; doi:10.1038/s41467-020-16145-4)
Supplement: Supplementary file 1 — Supplementary Information [file 41467_2020_16145_MOESM1_ESM.pdf]

Supplementary Information

Minimal lactazole scaffold for *in vitro* bioengineering of thiopeptides

Vinogradov et al

Contents

Supplementary Methods.. 2
Supplementary Figures.. 7
Supplementary References 39

Supplementary Methods

General. Reagents were purchased from Nacalai Tesque, Wako Pure Chemical Industries, Sigma-Aldrich Japan, Kanto Chemical, or Watanabe Chemical Industries unless noted otherwise and were used as received. Oligonucleotides were purchased from Eurofins Genomics (OPC purification grade) and were used without further purification. *E. coli* DH5α was used for plasmid manipulation. All PCR amplifications were carried out in a BioER TC-96GHBC thermal cycler. Sanger sequencing of constructed plasmids was done with appropriate primers by FASMAC (Tokyo, Japan).

Preparation of tRNA and flexizymes. tRNAs and flexizymes were prepared by *in vitro* transcription with T7 RNA polymerase from DNA templates encoding corresponding sequences downstream of a T7 promoter. Analogous to wild type *lazA*, DNA was assembled by PCR. Forward and reverse primers were extended, and then further amplified by PCR under the standard conditions. Primer sequences and assembly schemes can be found in Supplementary Data. PCR products were extracted by phenol/chloroform/isoamyl alcohol (25: 24: 1, saturated with 10 mM Tris (pH 8.0), 1 mM EDTA), chloroform/isoamyl alcohol (24: 1), and precipitated with ethanol. DNA were redissolved in water and added to the transcription reaction mix (40 mM Tris buffer (pH 8.0) supplemented with 22.5 mM $MgCl_2$, 10 mM DTT, 1 mM spermidine, 0.01% Triton X-100, 120 nM T7 RNA polymerase, 0.04 U/μL RNasin RNase inhibitor, and 3.75 mM each NTP). For tRNA transcription, 5 mM GMP was additionally supplied to the reaction mixture. Transcriptions were conducted at 37°C for 12-16 h on a 2 mL scale. After, 60 μL of 1 unit/μL RQ1 RNase-free DNase (Promega) was added, and the reactions were further incubated for 60 min at 37°C. The transcripts were precipitated with isopropanol, redissolved in water, and purified by 8% (tRNAs) or 12% (flexizymes) polyacrylamide gel containing 6 M urea. RNA extracted from the gel with 300 mM NaCl were collected by ethanol precipitation followed by centrifugation (15300 g for 15 min), and dissolved in water for storage.

tRNA Aminoacylation. For genetic code reprogramming experiments, $tRNA^{fMet}$, $tRNA^{GluE2}$ and $tRNA^{AsnE2}$ were aminoacylated with non-proteinogenic amino acids by the use of flexizymes. In general, 25 μM tRNA and 25 μM flexizyme were incubated with 5 mM activated amino acid ester (3,5-dinitrobenzyl or cyanomethyl esters; all prepared as previously established)[1] in 50 mM HEPES-KOH buffer (pH 7.5) containing 600 mM $MgCl_2$ on ice for 2 h. The reactions were stopped with the addition of 300 mM NaOAc (pH 5.2), and precipitated with ethanol. Precipitated RNA was recovered by centrifugation (15300 g for 15 min). The pellets were washed with 70% ethanol in water (v/v)

containing 100 mM NaOAc (pH 5.2) and used in translation. For some amino acids, conditions deviated from this general protocol; a complete list of aminoacylation conditions (flexizyme, activated ester, pH, and reaction time) can be found in Supplementary Data.

Design of randomized sequences. Randomized peptide sequences were generated with ExPASy RandSeq tool (https://web.expasy.org/randseq/), using average amino acid compositions computed from Swiss-Prot. Peptides containing Cys, overly repetitive or hydrophobic sequences were discarded. Negatively charged amino acids in positions 3, 4, 11 and 12 (sites adjacent to the residues undergoing PTM), and positively charged amino acids in positions 11 and 12 were avoided as well.

***In vitro* translation with genetic code reprogramming**. Due to the presence of a 38-residue LP in LazA composed of 16 kinds of proteinogenic amino acids, the codon boxes available for reprogramming are limited to His, Lys, Phe and Tyr codons. Additionally, the AUG Met codon can be utilized if translation initiation is also reprogrammed, and the UGG Trp codon is available if a mutation of Trp2 in the CP is deemed acceptable. We chose the AUG codon for incorporation of single npAAs, and UGG, CAU, AAG and UUU codons for synthesizing a thiopeptide with 4 npAAs.

Genetic code reprogramming experiments generally followed the workflow from above, except aminoacylated tRNA replaced specific proteinogenic amino acids corresponding to the reprogrammed codons during translation. For incorporation of single npAAs, 50 µM *N*-biotinylated-Phe-$tRNA^{fMet}_{CAU}$ and 50 µM npAA-$tRNA^{GluE2}_{CAU}$ were added to the translation mixture that lacked Met and 10-HCO-H4 folate. As a result, peptides bearing *N*-biotinylated-Phe instead of formyl-Met, and an npAA instead of Met were expressed. Analogously, expression from a Lys/His/Phe/Trp-depleted translation reaction supplemented with ^{Me}Gly-$tRNA^{GluE2}_{CUU}$, ^{Me}Ala-$tRNA^{GluE2}_{AAA}$, cLeu-$tRNA^{GluE2}_{GUG}$, and Phe(F_5)-$tRNA^{AsnE2}_{CCA}$ resulted in the peptide containing 4 npAAs of interest. In this case, the concentrations of exogenously added tRNA were adjusted to 25 µM each.

Thiopeptide quantification. Quantification of thiopeptides synthesized with the FIT-Laz system was performed in two ways. First, the amount of produced lactazole A was directly measured against an LC-MS standard calibration curve generated with a sample of the authentic thiopeptide. Additionally, a method relying on an LC-MS standard curve generated for the second product of the final macrocyclization step, LP-NH_2 was utilized as an indirect quantification approach; synthetic LP-NH_2 of a known concentration was used as a standard in this case. The latter quantification method enabled a semi-quantitative estimation of thiopeptide yields produced from LazA variants.

Synthesis and purification of LP-NH_2.

The peptide (primary sequence: SDITASRVESLDLQDLDLSELTVTSLRDTVALPENGA) was synthesized using standard batch Fmoc SPPS protocols on a Syro Wave automated peptide synthesizer (Biotage). NovaPEG Rink Amide resin (0.44 mmol/g loading, 6 x 55 mg) was used to synthesize the peptide as a C-terminal amide. Side chain protection was as follows: Asn(Trt), Asp(OtBu), Gln(Trt), Glu(OtBu), Lys(Boc), Ser(tBu), Thr(tBu), Arg(Pbf); Fmoc protected amino acids were purchased from Novabiochem or Watanabe Chemical Industries. Peptide couplings were performed with (2-(1H-benzotriazol-1-yl)-1,1,3,3-tetramethyluronium hexafluorophosphate (HBTU) as an activating agent and diisopropylethylamine (DIPEA) as a base in dimethylformamide (DMF); double coupling (1 h each) was performed at every residue. Fmoc removal was done with a 20% (v/v) solution of piperidine in DMF. After peptide assembly, the resin was washed with dichloromethane (DCM) three times, and with DMF twice. Formylation was performed manually: to a solution of 4-nitrophenylformate in DMF (400 mM; 3.75 mL), DIPEA (3 eq.) was added, and the mixture was transferred to the peptidyl resin. After 1 h, the resin was thoroughly washed with DMF, and the formylation step was repeated once allowing the reaction to proceed overnight. After washing the resin with DMF (three times) and DCM (three times), the peptide was cleaved from the solid support with 8 mL trifluoracetic acid/water/ethanedithiol/triisopropylsilane (94/2.5/2.5/1.0, v/v) cocktail for 2 h at room temperature. Deprotected peptide was precipitated with 40 mL cold diethyl ether, and the supernatant was separated by centrifugation (5 min, 5000g). The pellet was washed with cold ether three more times, after which the peptide was resuspended in 20 mL of water/acetonitrile (30/70, v/v) containing 0.1% TFA, sonicated and lyophilized to dryness. Crude lyophilized peptide was dissolved in 8 mL of dimethyl sulfoxide (DMSO), filtered and purified by reverse phase HPLC on a Shimadzu LC-20AP instrument equipped with a Chromolith Prep column (100 x 25 mm, C18 phase; Merck) using 0.1% TFA in water (solvent A') and 0.1% TFA in acetonitrile (solvent B') as a mobile phase at 20 mL/min flow rate. The following gradient was used: 10% B' for 5min; 10 to 25% B' over 15 min; 25 to 45% B' over 40 min. LP-NH_2 eluted at around 38% B under these conditions. Fractions containing pure LP-NH_2 were combined and lyophilized, yielding 7.0 mg peptide (Supplementary Fig. 1).

Generation of LC-MS calibration curves for lactazole A and LP-NH_2. A 10-point calibration curve was generated for lactazole A. Solution of the authentic thiopeptide in DMSO (1 mM) was diluted with a 1/1 (v/v) mixture of methanol and reaction buffer to make analytes ranging in concentration from 0.013 μM to 1.34 μM. The samples were analyzed by HPLC-MS on a C4 column

as specified in Methods. Instrument response was measured as the total area under the peak, and the calibration curve was generated from linear regression of instrument response to lactazole A injection amount (Supplementary Fig. 2a).

For LP-NH_2, lyophilized HPLC-purified peptide was dissolved in DMSO to the final concentration of 50 mM, and diluted with a 1/1 (v/v) mixture of methanol and reaction buffer to make analytes ranging in concentration from 0.004 μM to 0.91 μM (11 samples). HPLC-MS analysis was performed similar to lactazole A, except area under the z=3 peak (*m/z* 1368.70) was used for calibration, as it gave higher dynamic range than total peak area. Linear regression of response vs. LP-NH_2 injection amount is shown in Supplementary Fig. 2b.

Quantification of thiopeptide production in FIT-Laz. LazA and $LazA^{min}$ were expressed and modified in the FIT-Laz system as described in Methods, except the biosynthesis incubation time was shortened to 5 h. HPLC-MS analysis was performed on a C4 column following the conditions described in Methods.

For LazA, the amounts of produced lactazole A and LP-NH_2 were calculated from the calibration curves shown in Supplementary Fig. 2. A 2.5 μl translation reaction resulted in 18.7 ± 0.4 pmol (± st. dev. from three experiments) lactazole A, and 21 ± 2 pmol LP-NH_2 (Supplementary Fig. 3), which corresponds to 1.24 ± 0.02 μM lactazole A and 1.4 ± 0.1 μM LP-NH_2 in the reaction mixture at the time of quenching, or to 7.5 ± 0.2 μM (calculation based on the amount of lactazole A) and 8.4 ± 0.6 μM (calculation based on the amount of LP-NH_2) of LazA precursor peptide (which underwent productive conversion to the thiopeptide/LP-NH_2 pair) at the end of the translation reaction. These results indicate that a) lactazole A biosynthesis in the FIT-Laz system proceeds with high efficiency, considering that ~8-10 μM translation yield represents the practical upper limit achievable with reconstituted *in vitro* translation systems;[2] b) thiopeptide and LP-NH_2 formation are coupled to a large degree, suggesting that the thiopeptide production efficiency can be gauged from quantifying formation of LP-NH_2 and by analyzing ^{br}EIC chromatograms (in a qualitative manner as described in Methods).

For $LazA^{min}$, quantification was performed from the amount of produced LP-NH_2. In this case, 18 ± 2 pmol LP-NH_2 formed (1.2 ± 0.1 μM at the time of quenching; 7.1 ± 0.9 μM $LazA^{min}$ at the end of the translation reaction), indicating that the maturation of $LazA^{min}$ in FIT-Laz proceeds nearly as efficiently as LazA.

For other thiopeptides, the amount of formed LP-NH_2 was *estimated* using the same calibration

curve (Supplementary Data). In these cases, the data are intended to serve as a semi-quantitative estimation rather than direct quantification for three reasons:

i) As data were acquired over a period of time, drift of the mass spectrometer detector sensitivity over time contributes to quantification uncertainty;

ii) Quantification of LP-NH_2 levels does not control for peptide expression level, and as such, numerical comparisons between different peptides may be of limited utility;

iii) In some cases, as discussed where appropriate, the Met AUG initiation codon was genetically reprogrammed to biotin-Phe, which resulted in the production of LP-NH_2 of a slightly different chemical structure. Quantification of LP^*-NH_2 was done under the assumption that the mutation does not affect the peptide's ionization efficiency.

Supplementary Figures

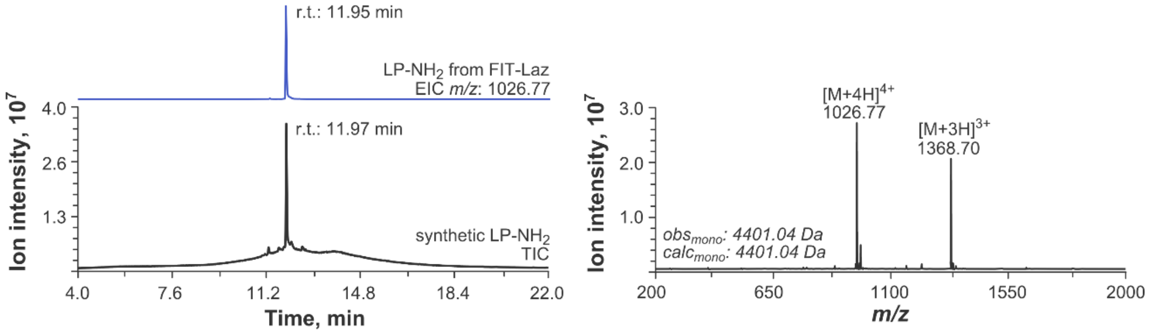

Supplementary Figure 1. HPLC-MS (TIC) chromatogram for the purified synthetic LP-NH_2 on the left with a mass spectrum of the major peak on the right. HPLC retention time of the synthetic peptide matches that of LP-NH_2 produced with the FIT-Laz system. An EIC chromatogram (*m/z* 1026.77 ± 1.00) for LP NH_2 produced from the FIT-Laz system is displayed for comparison.

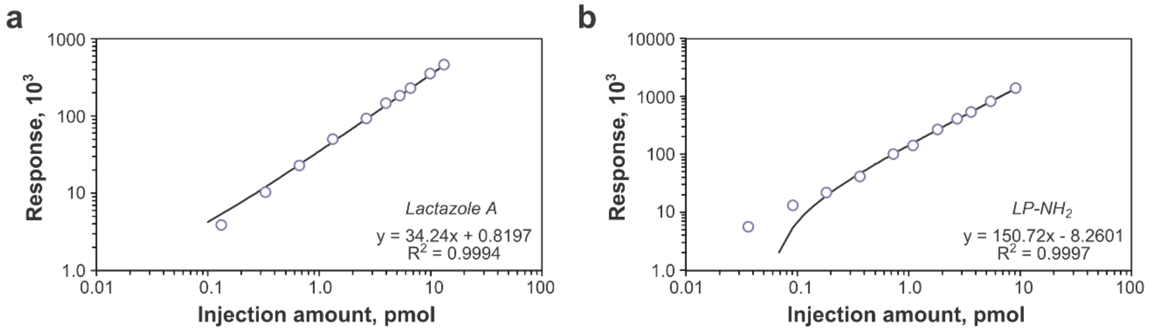

Supplementary Figure 2. HPLC-MS calibration curves for lactazole A (**a**) and LP-NH_2 (**b**). For lactazole A, response corresponds to the total area under the peak; for LP-NH_2 – area under the z=3 peak. Note, that for visualization purposes, log_{10}-log_{10} plots are shown; however, the data was fit using least squares linear regression as the inset equations indicate.

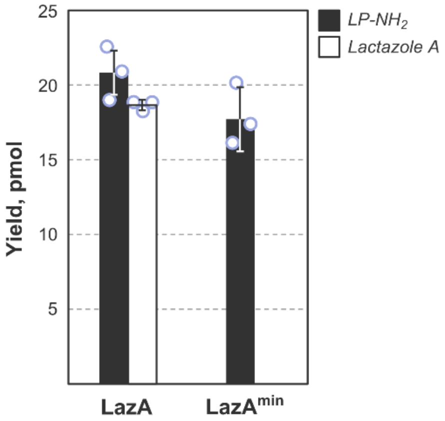

Supplementary Figure 3. Quantification of the amounts of lactazole A and LP-NH_2 produced from a 2.5 µl translation reaction in the FIT-Laz system. Bar plot data are presented as mean measured values (n = 3; independent experiments) ± one standard deviation. Individual measurements are displayed for reference.

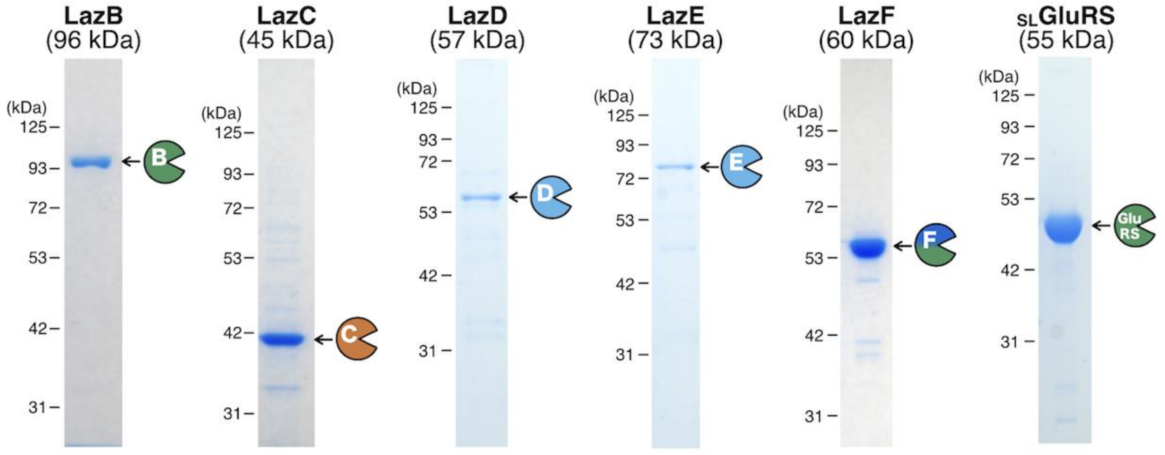

Supplementary Figure 4. SDS-PAGE analysis of recombinantly produced lactazole biosynthetic enzymes utilized in this work. For protein expression details refer to the Methods section. Each enzyme was purified at least twice throughout the course of the work to a similar result.

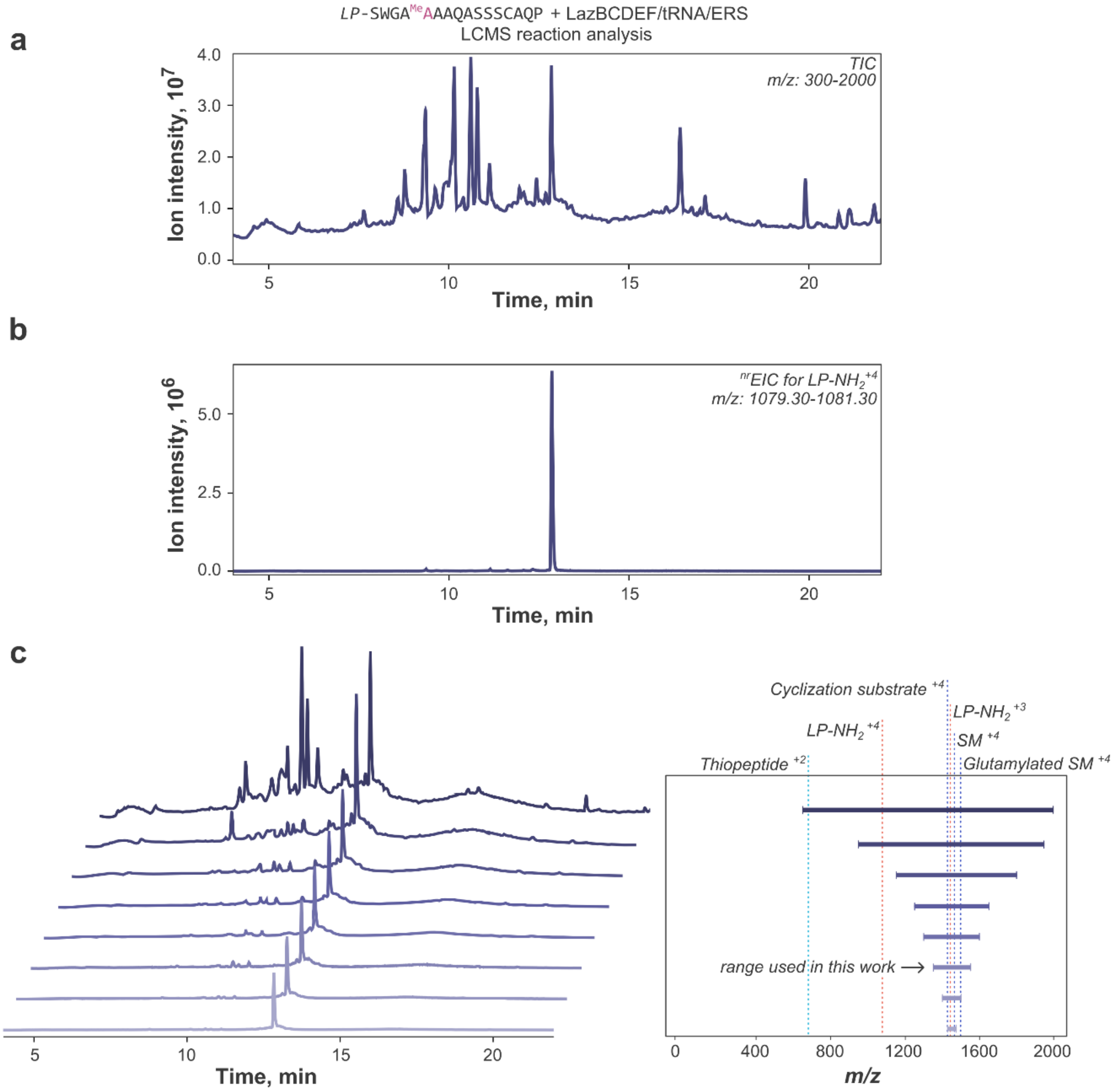

Supplementary Figure 5. An illustration to the data analysis routines and the ^{br}EIC concept described in Methods. **(a)** TIC chromatogram for the reaction between a LazA^{min} variant (CP sequence: SWGA$^{\mathbf{Me}}$**A**AAQASSSCAQP) and Laz enzymes. A number of translation components obfuscate interpretation of the chromatogram. **(b)** EIC at *m/z* 1080.30±1.00 corresponding to LP*-NH_2 produced during the final macrocyclization reaction. LP* stands for LazA LP sequence where formyl-Met is replaced with N-biotinylated-Phe (see Supplementary Methods for details). **(c)** A visual explanation of the ^{br}EIC concept. With the exception of the final thiopeptide, all linear intermediates and side-products cluster around m/z ~1400, whereas translation components are characterized by lower *m/z* values. Sequential generation of EIC chromatograms with narrower and narrower *m/z* windows eliminates more and more translation components unrelated to the reaction in question. EIC chromatograms at *m/z*(precursor peptide) ± 100 capture all LazA^{min}-derived products with the exception of the mature thiopeptide, for which a separate, narrow range chromatogram was generated in each case.

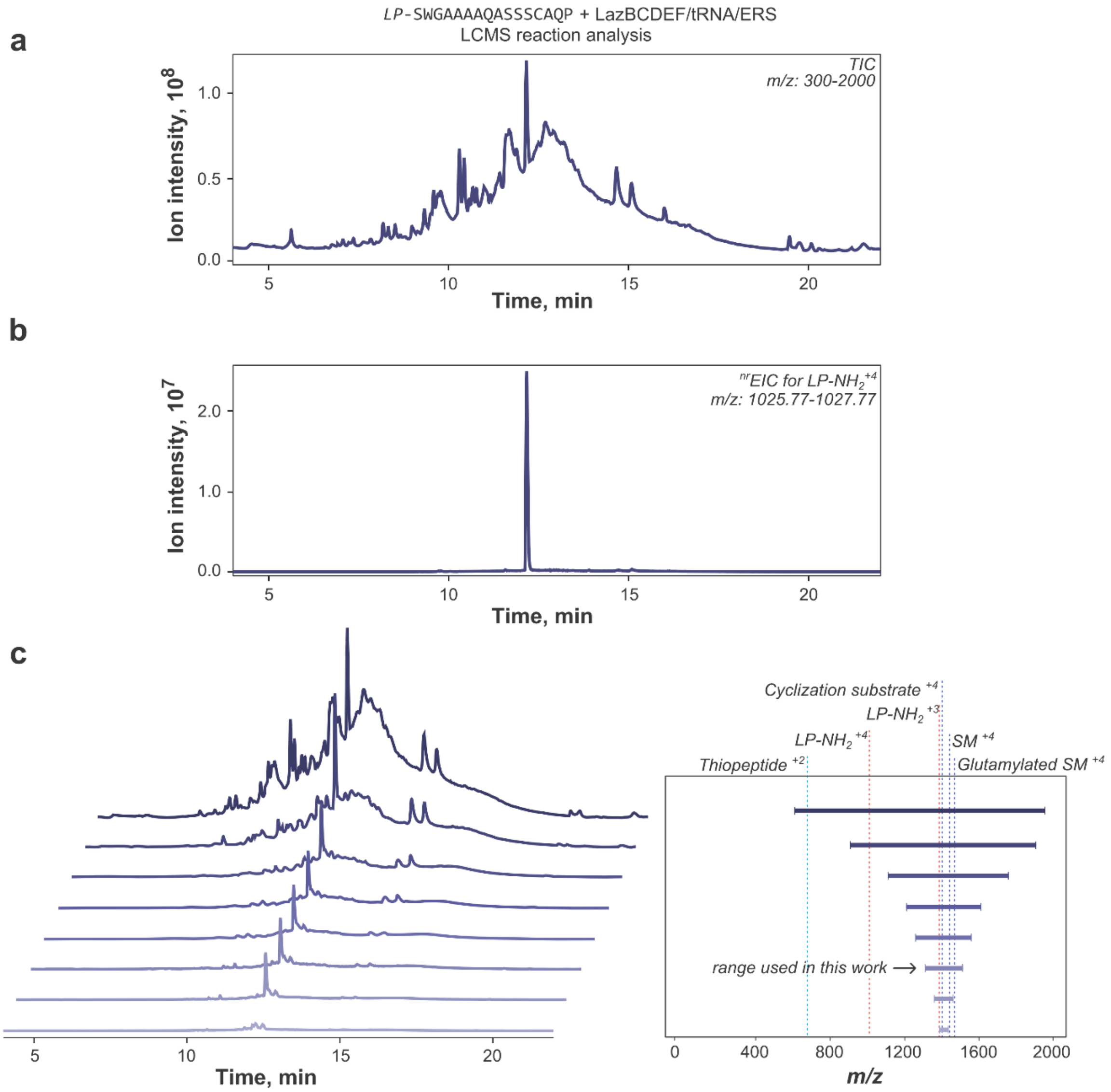

Supplementary Figure 6. An illustration to the data analysis routines and the ^{br}EIC concept described in Methods. **(a)** TIC chromatogram for the reaction between LazA^{min} and Laz enzymes. A number of translation components obfuscate interpretation of the chromatogram. **(b)** EIC at *m/z* 1026.77±1.00 corresponding to LP-NH$_2$ produced during the final macrocyclization reaction. **(c)** A visual explanation of the ^{br}EIC concept. With the exception of the final thiopeptide, all linear intermediates and side-products cluster around m/z ~1400, whereas translation components are characterized by lower *m/z* values. Sequential generation of EIC chromatograms with narrower and narrower *m/z* windows eliminates more and more translation components unrelated to the reaction in question. EIC chromatograms at *m/z*(precursor peptide) ± 100 capture all LazA^{min}-derived products with the exception of the mature thiopeptide, for which a separate, narrow range chromatogram was generated in each case.

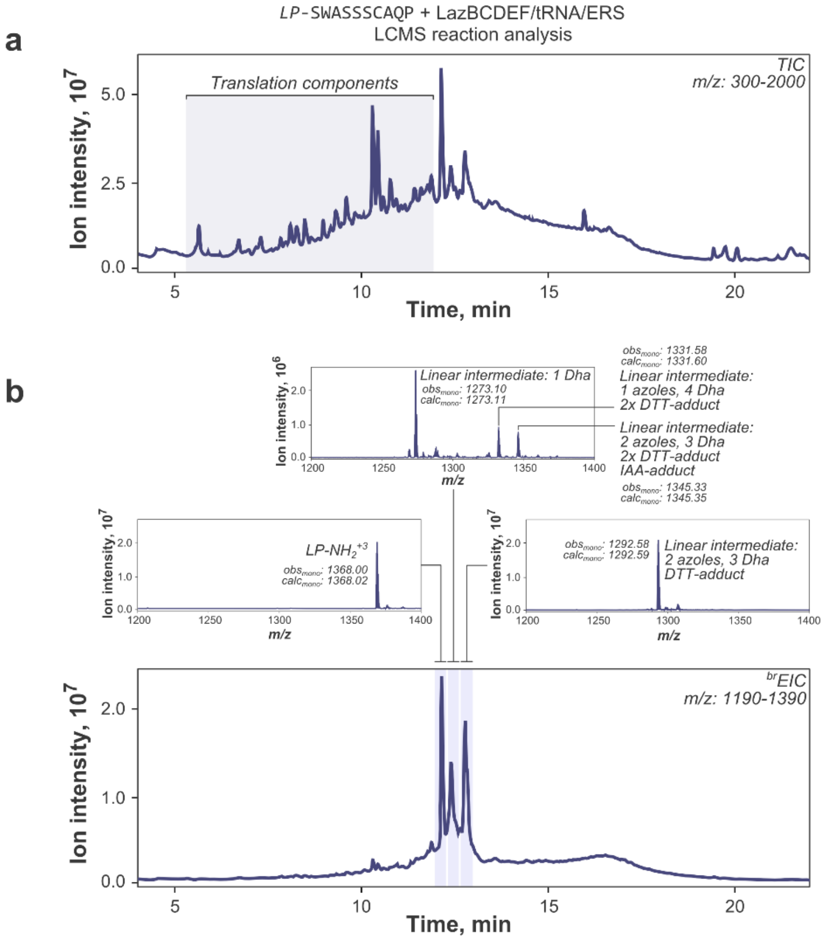

Supplementary Figure 7. An example of LC-MS data analysis performed with the use of ^{br}EIC. **(a)** TIC chromatogram for the reaction between a LazA^{min} variant (CP sequence: SWASSSCAQP) and Laz enzymes. A number of translation components obfuscate interpretation of the chromatogram. **(b)** ^{br}EIC chromatogram generated at *m/z* 1290 ± 100 with MS insets integrated over shaded regions. The resulting ^{br}EIC chromatogram isolates LazA-derived products from translation components, simplifying interpretation and analysis. In this case, the product indicative of the thiopeptide formation ($LP-NH_2$; the leftmost peak) is accompanied by a number of identifiable linear intermediates and side-products.

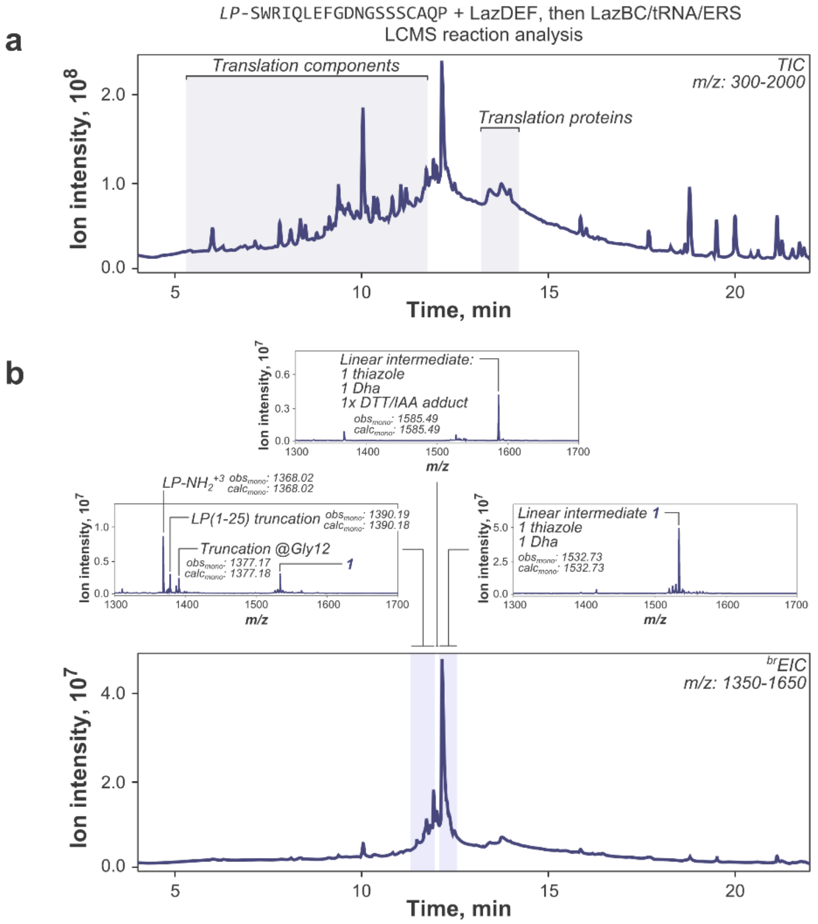

Supplementary Figure 8. An example of LC-MS data analysis performed with the use of brEIC. **(a)** TIC chromatogram for the reaction between a LazA^{min} variant (CP sequence: SWRIQLEFGDNGSSSCAQP) and Laz enzymes. A number of translation components obfuscate interpretation of the chromatogram. **(b)** brEIC chromatogram generated at *m/z* 1500 ± 150 with MS insets integrated over shaded regions. The resulting chromatogram isolates LazA-related products from translation components, simplifying interpretation and analysis. In this case, a minute amount of LP-NH_2 is accompanied by a number of linear intermediates, side-products and sequence truncations.

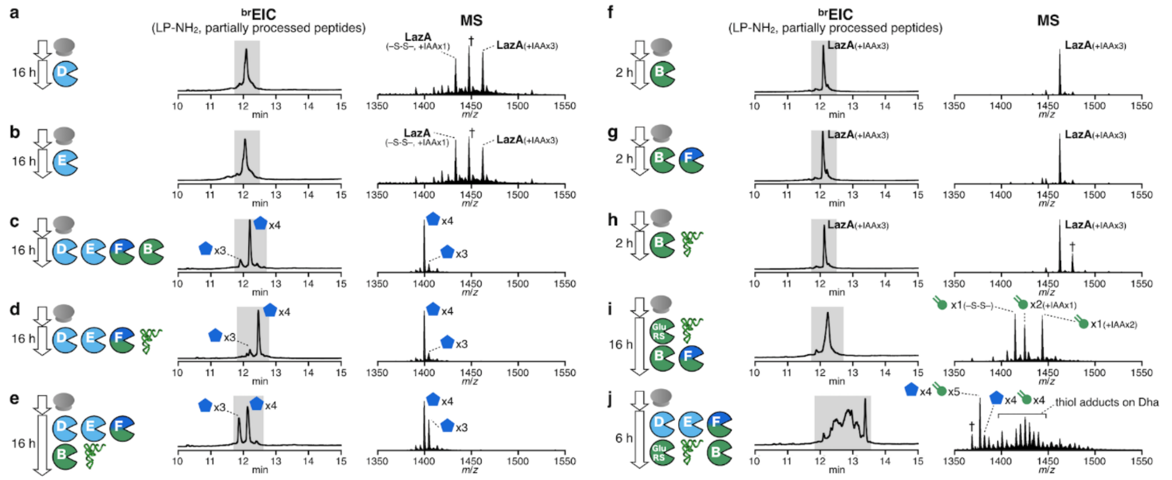

Supplementary Figure 9. Reconstitution of *in vitro* lactazole A biosynthesis. **(a)** – **(j)** Reconstitution of azole and Dha formation in FIT-Laz. LazA precursor peptide produced with the FIT system was treated with a combination of Laz enzymes as indicated in each panel and the reaction outcomes were analyzed by LC-MS. Displayed are ^{br}EIC LC-MS chromatograms and composite mass spectra integrated over a time period shaded in the corresponding chromatograms. See Methods and Supplementary Figs. 5–8 for details on reaction conditions and the explanation of ^{br}EIC chromatograms. MS peaks labeled with † are unidentified products.

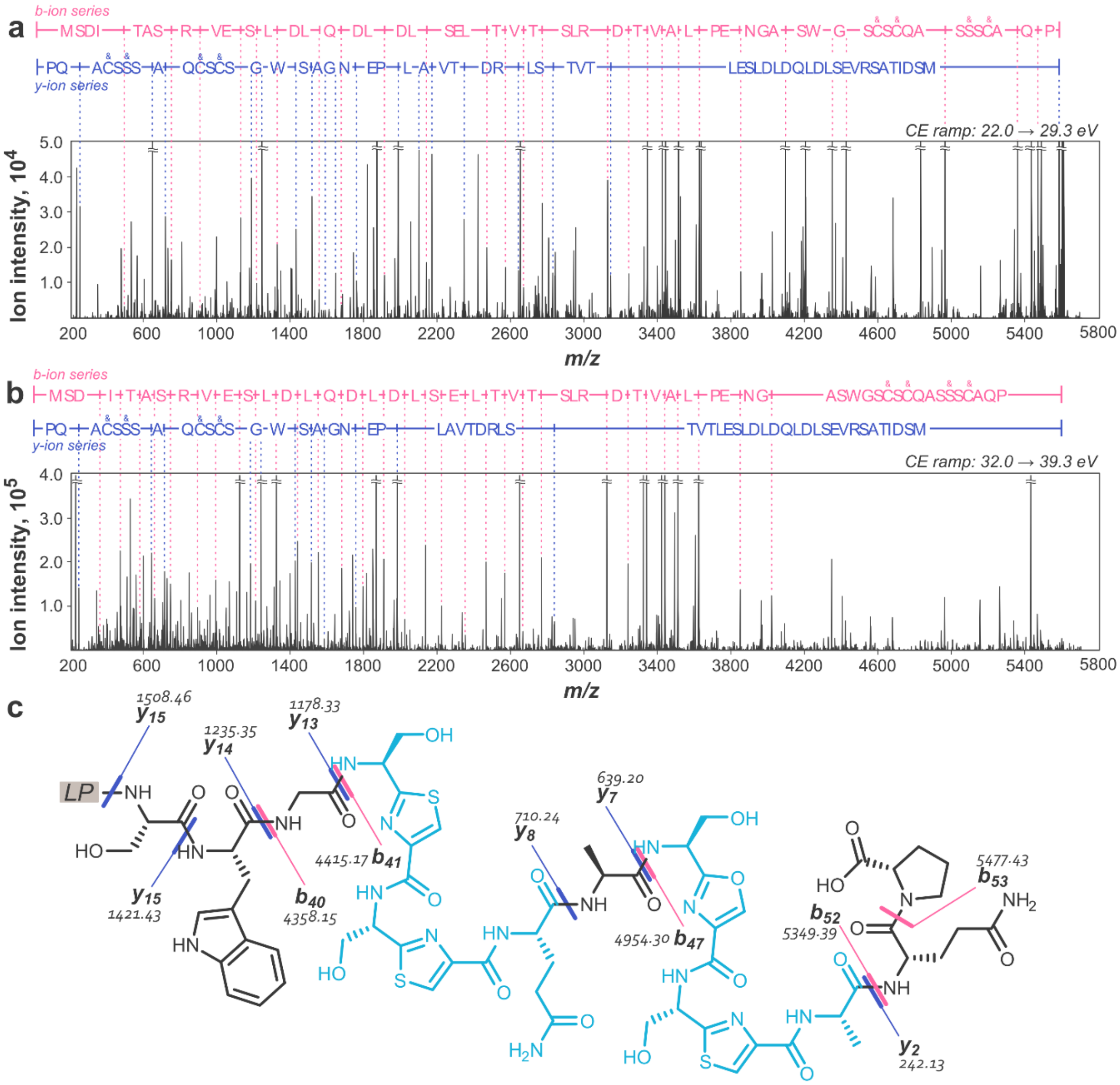

Supplementary Figure 10. MS/MS spectrum of LazA after treatment with LazDEF. **(a)** Charge-deconvoluted CID fragmentation spectrum obtained with collision energies ramped from 22.0 to 29.3 eV and spectral assignments; *y*- and *b*-ions are annotated; stable molecule losses (H_2O, NH_3, CO, etc) and double fragmentation assignments are omitted for clarity; ampersands denote azole formations; “fM” stands for formyl-methionine; **(b)** Analogous to (a) except the spectrum was obtained with collision energies ramped from 32.0 to 39.3 eV. c) Assigned chemical structure of the LazA CP region with mapped annotations. Each of the two regions highlighted in cyan contains 2 azole modifications, but the precise azole location could not be unambiguously assigned based on these spectra.

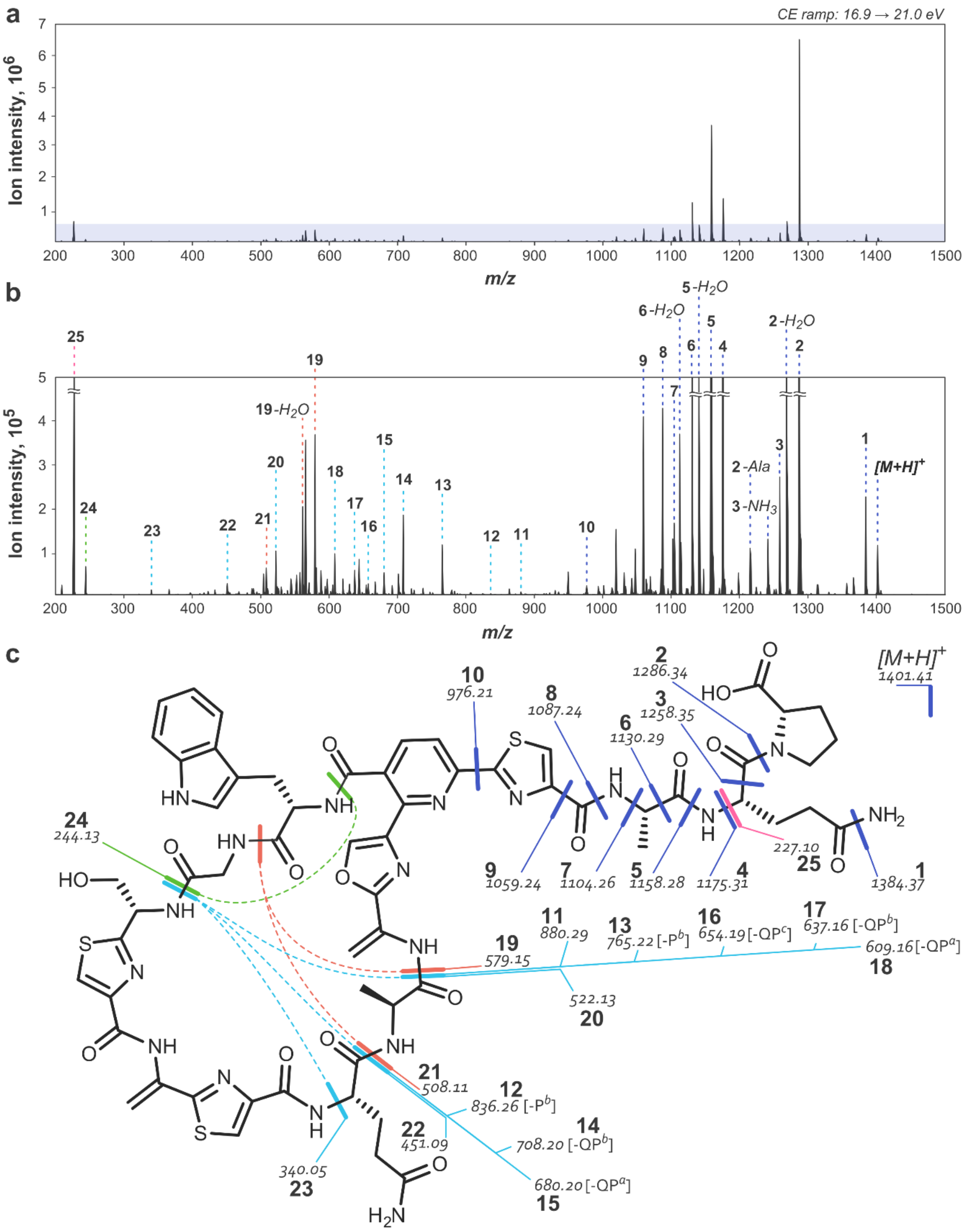

Supplementary Figure 11. Annotated fragmentation spectrum of lactazole A. **(a)** Charge-deconvoluted CID fragmentation spectrum obtained with collision energies ramped from 16.9 to 21.0

eV. **(b)** Y-axis zoom of the shaded area from (a) with spectral assignments. **(c)** Assigned chemical structure of lactazole A with mapped assignments. Under acquisition conditions the thiopeptide underwent multiple double fragmentations allowing the mapping of amino acids within the macrocycle. A number of triple fragmentations are annotated in cyan; for such assignments, positions of the third fragmentation in the tail region are indicated next to the corresponding *m/z* values. Some ions can have multiple potential assignments. In such cases, only 1 isomer is shown.

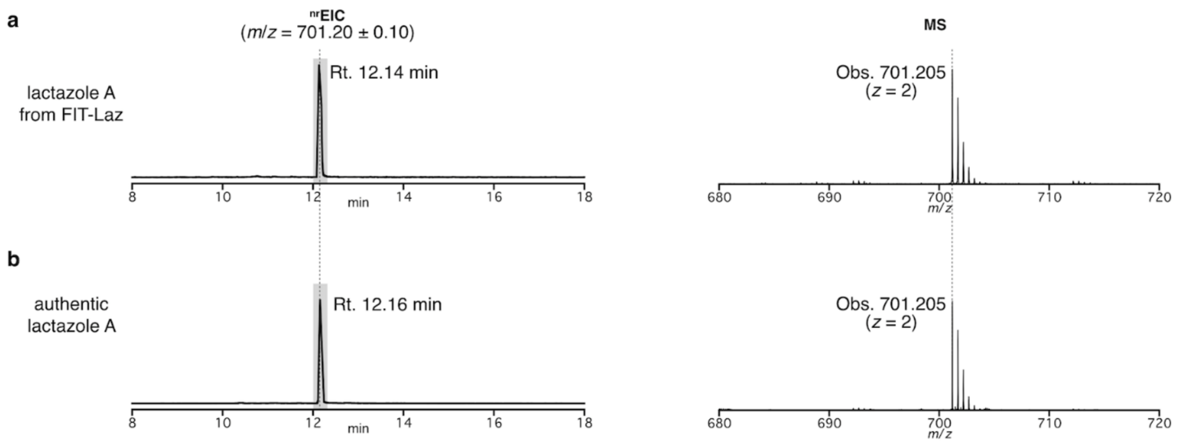

Supplementary Figure 12. LC-MS comparison between authentic lactazole A and lactazole A synthesized with the FIT-Laz system. Left: ^{nr}EIC chromatograms generated at m/z 701.20 ± 0.10; right: composite mass spectra integrated over a time period shaded in the corresponding chromatograms. **a)** Data for lactazole A produced with the FIT-Laz system. **b)** Data for authentic lactazole A.

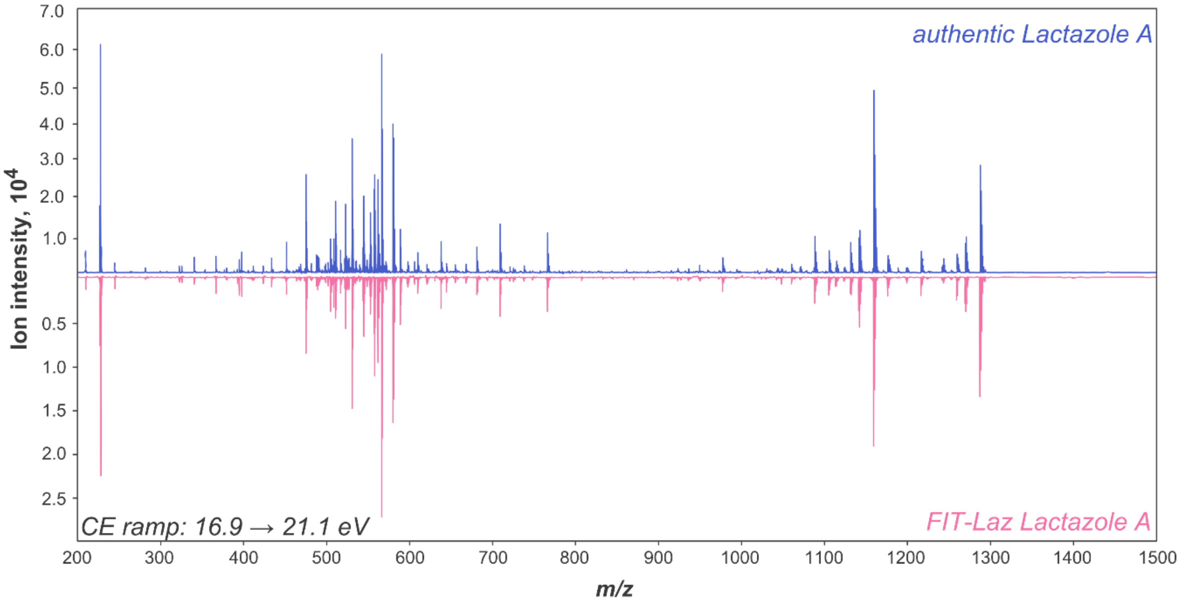

Supplementary Figure 13. Overlaid CID fragmentation spectra of authentic lactazole A (blue spectrum) and lactazole A synthesized with the FIT-Laz system (red). Identical fragmentation patterns confirm authenticity of the synthesized thiopeptide.

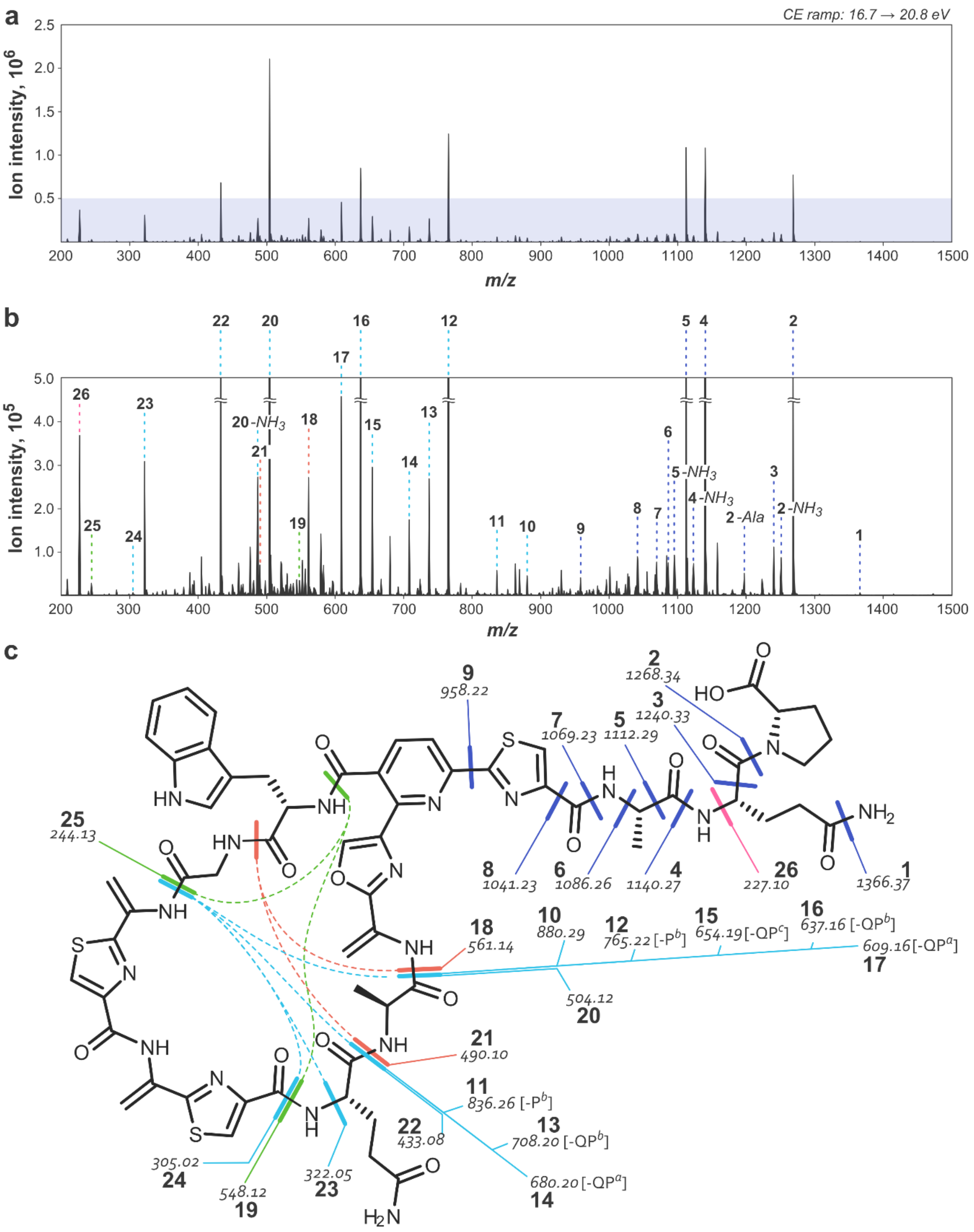

Supplementary Figure 14. Annotated fragmentation spectrum of Dha4-lactazole synthesized with the stepwise enzyme treatment. **(a)** Charge-deconvoluted CID fragmentation spectrum obtained with

collision energies ramped from 16.7 to 20.8 eV. **(b)** Y-axis zoom of the shaded area from (a) with spectral assignments. **(c)** Assigned chemical structure of Dha4-lactazole with mapped assignments. Under acquisition conditions the thiopeptide underwent multiple double fragmentations allowing the mapping of amino acids within the macrocycle. A number of triple fragmentations are annotated in cyan; for such assignments, positions of the third fragmentation in the tail region are indicated next to the corresponding *m/z* values. Some ions can have multiple potential assignments. In such cases, only 1 isomer is shown. Extra dehydration compared to lactazole A can be unambiguously localized between residues 4 and 7. Because numerous fragments originating at Gly3-Ser4 bond were observed, dehydration to Dha rather than oxazoline formation at Ser4 can be deduced.

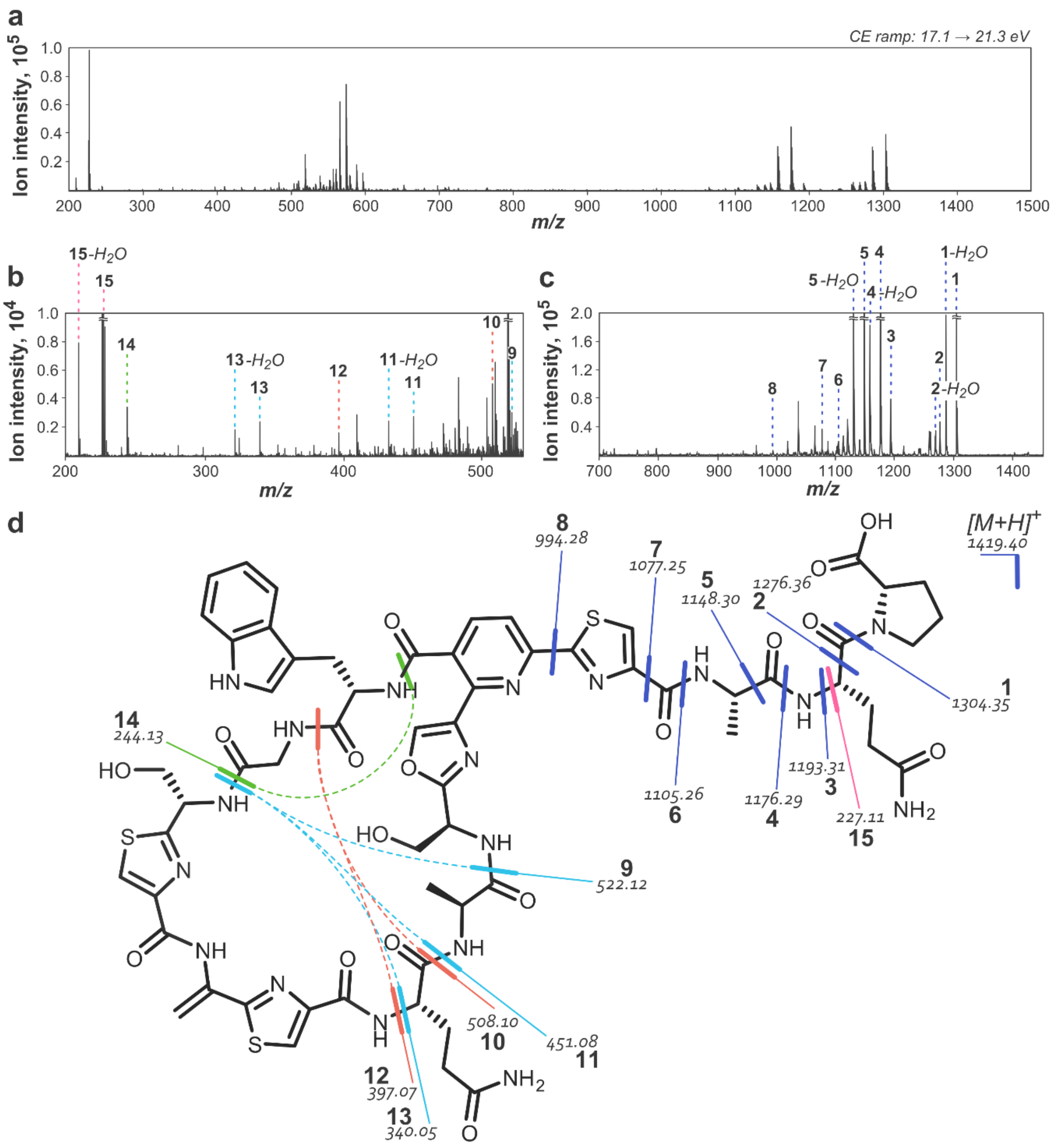

Supplementary Figure 15. Annotated fragmentation spectrum of Ser10-lactazole synthesized with the stepwise enzyme treatment. **(a)** Stacked CID fragmentation spectrum (total acquisition time: 12 s) obtained with collision energies ramped from 17.1 to 21.3 eV. **(b)** Y-axis zoom of the low mass region of the spectrum (*m/z* 200 to 530) with spectral assignments. **(c)** Y-axis zoom of the high mass region of the spectrum (*m/z* 700 to 1500) with spectral assignments. **(d)** Assigned chemical structure of Ser10-lactazole with mapped assignments. In contrast to lactazole A and Dha4-lactazole, Ser10-

lactazole proved to be significantly more resistant to fragmentation inside the macrocycle, which necessitated spectral stacking in order to observe key fragments. The tail fragmentation pattern (blue/pink) is nearly identical to lactazole A, supporting the same structural assignment. Fragments 9-13 indicate that residues 4-7 have native modification pattern, suggesting that Dha → Ser replacement takes place at residue 10. Some ions can have multiple potential assignments. In such cases, only 1 isomer is shown. A number of annotations is omitted for clarity.

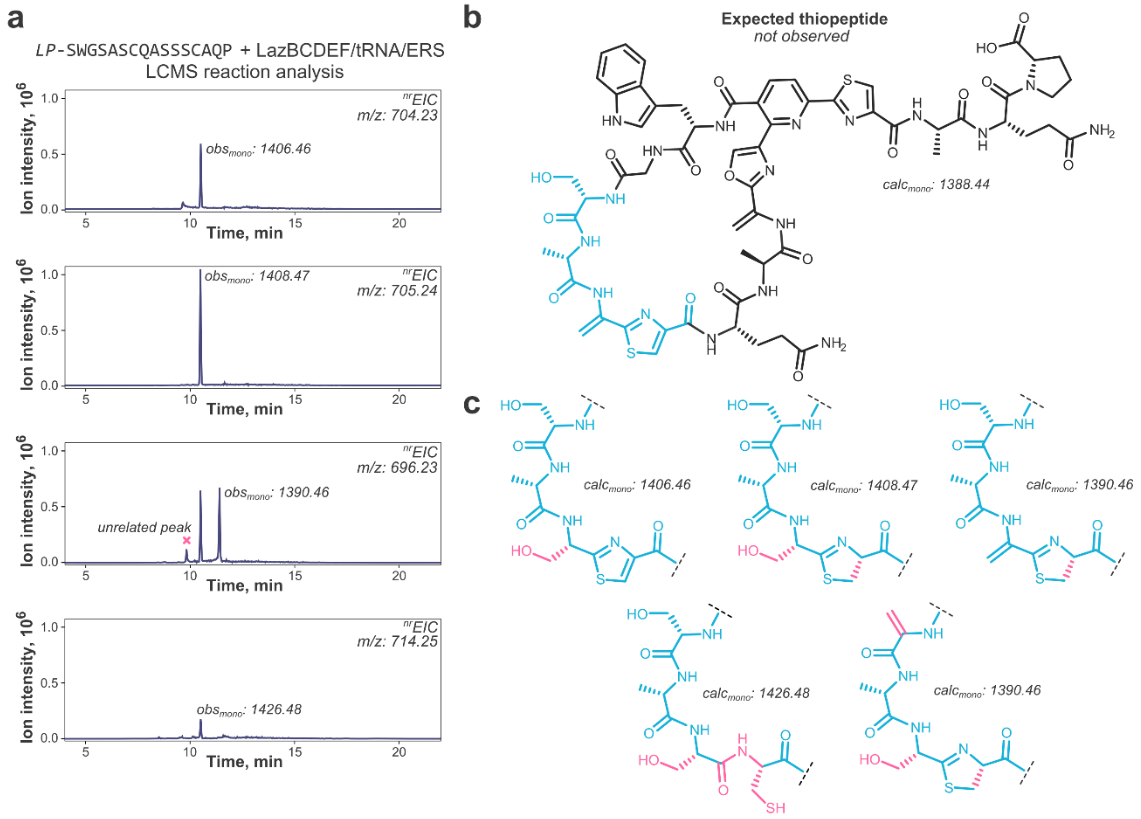

Supplementary Figure 16. Different thiopeptides generated in the FIT-Laz system for LazA C5A mutant. **(a)** ^{nr}EIC chromatograms generated at *m/z* 0.10 tolerance windows for detected thiopeptides; a total of 5 thiopeptides was observed. **(b)** Chemical structure of the expected lactazole mutant. In this case, the expected product was not detected. The region highlighted in cyan indicates a region with a divergent pattern of PTMs. **(c)** Plausible chemical structures of observed thiopeptides. The constant region is omitted for clarity. Differences between the expected and assigned structures are highlighted in pink.

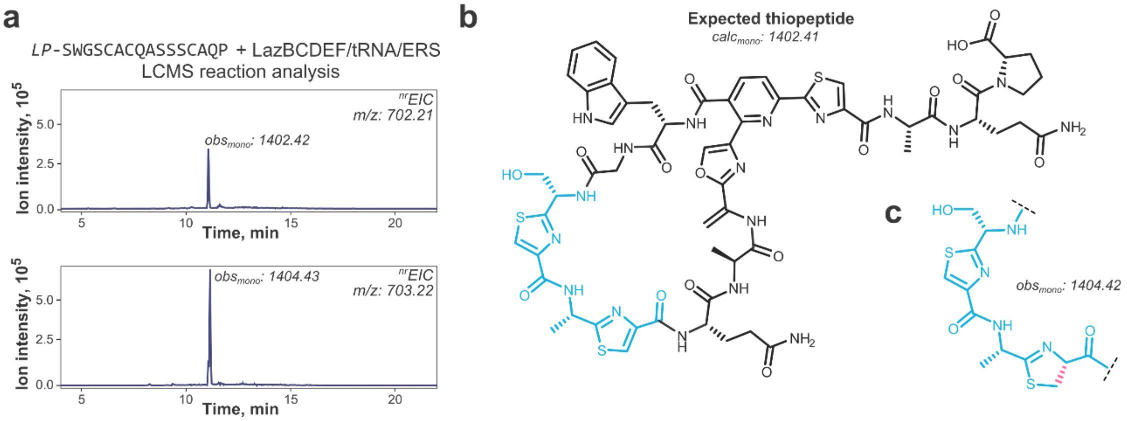

Supplementary Figure 17. Different thiopeptides generated in the FIT-Laz system for LazA S6A mutant. **(a)** ^{nr}EIC chromatograms generated at *m/z* 0.10 tolerance windows for detected thiopeptides; 2 thiopeptides were observed. **(b)** Chemical structure of the expected lactazole mutant. The region highlighted in cyan indicates a region with a divergent pattern of PTMs. **(c)** Plausible chemical structure observed for the second thiopeptide. The constant region is omitted for clarity. Differences between the expected and assigned structures are highlighted in pink.

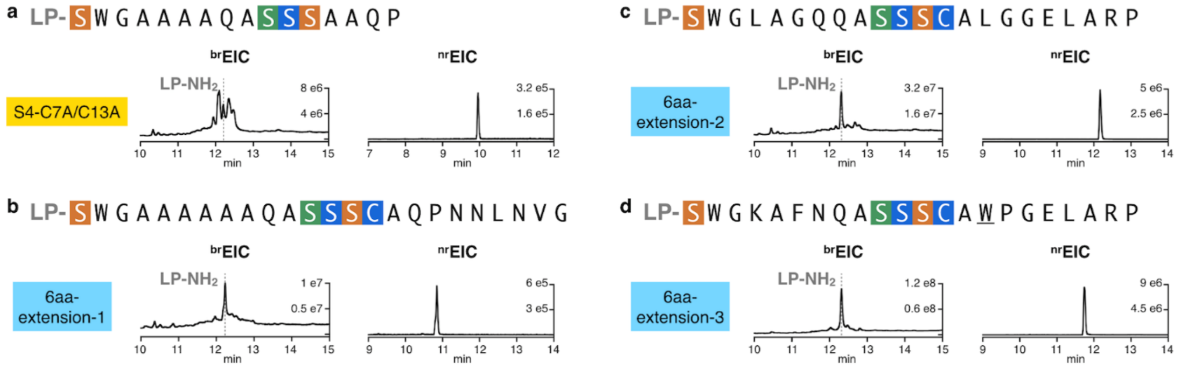

Supplementary Figure 18. Substrate scope of the FIT-Laz system. **(a)** – **(d)** Variants of LazA^{min} were treated with the full enzyme set and the outcomes were analyzed by LC-MS. Displayed are LC-MS chromatograms (^{br}EIC chromatograms on the left showing partially processed linear peptides and LP-NH_2 after enzymatic treatment, and ^{nr}EIC chromatograms on the right for expected thiopeptides generated at *m/z* 0.10 tolerance window). For mutants highlighted in light blue biosynthesis proceeded efficiently; yellow highlighting indicates inefficient thiopeptide formation accompanied by the accumulation of linear intermediates and side-products.

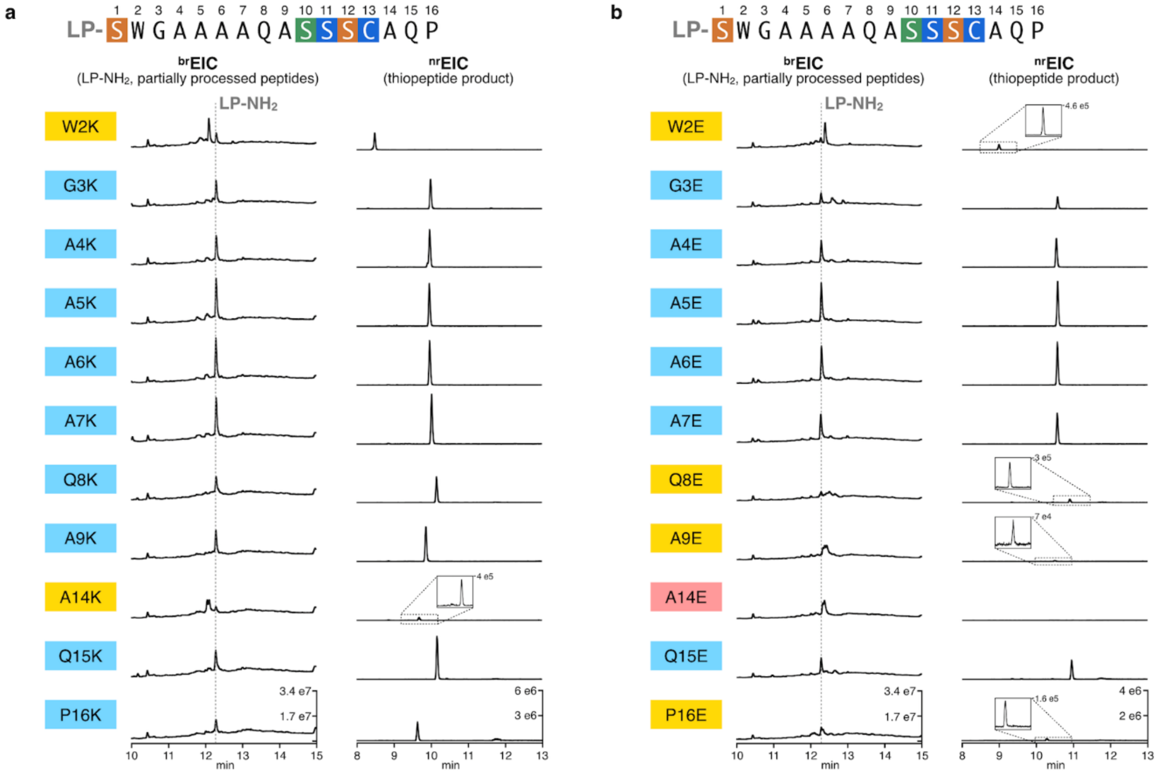

Supplementary Figure 19. Tolerance of Laz enzymes towards charged amino acids in the CP of LazA^{min}. **(a)** Lys-scanning and **(b)** Glu-scanning mutagenesis of LazA^{min}. Precursor peptides were treated with the full enzyme set and the outcomes were analyzed by LC-MS. Displayed are LC-MS chromatograms (^{br}EIC chromatograms on the left showing partially processed linear peptides and LP-NH_2 after enzymatic treatment, and ^{nr}EIC chromatograms on the right for expected thiopeptides generated at *m/z* 0.10 tolerance window). For mutants highlighted in light blue biosynthesis proceeded efficiently; yellow highlighting indicates inefficient thiopeptide formation accompanied by the accumulation of linear intermediates and side-products; red – mutants that failed to yield a detectable thiopeptide.

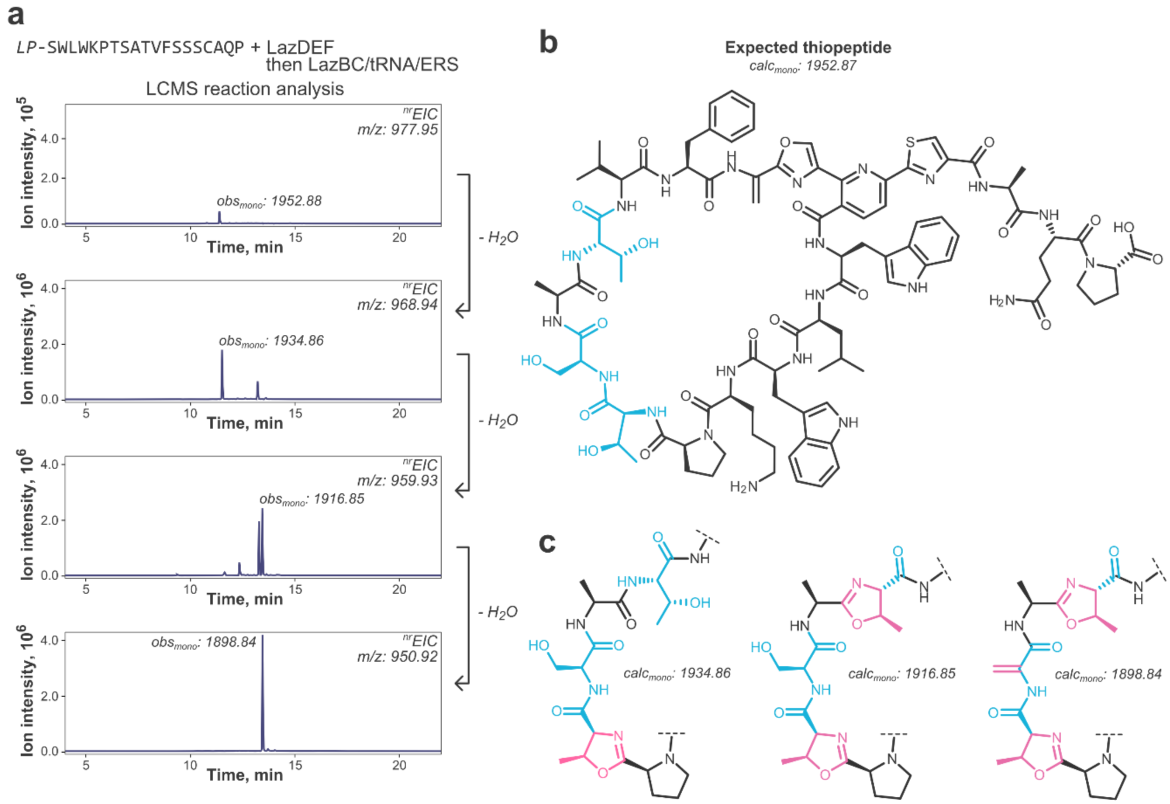

Supplementary Figure 20. Different thiopeptides generated in the FIT-Laz system for LazA^{min} 10aa-sub4. **(a)** ^{nr}EIC chromatograms generated at *m/z* 0.10 tolerance windows for detected thiopeptide; a total of 8 thiopeptides, differing in their dehydration patterns was observed. **(b)** Chemical structure of the expected thiopeptide. The region highlighted in cyan indicates residues may undergo dehydration in the FIT-Laz system. **(c)** Three plausible chemical structures for sequentially dehydrated thiopeptides. The constant region is omitted for clarity. Differences between the expected and assigned structures are highlighted in pink. Other isomers may arise due to the positional and/or Dha/oxazoline isomerism.

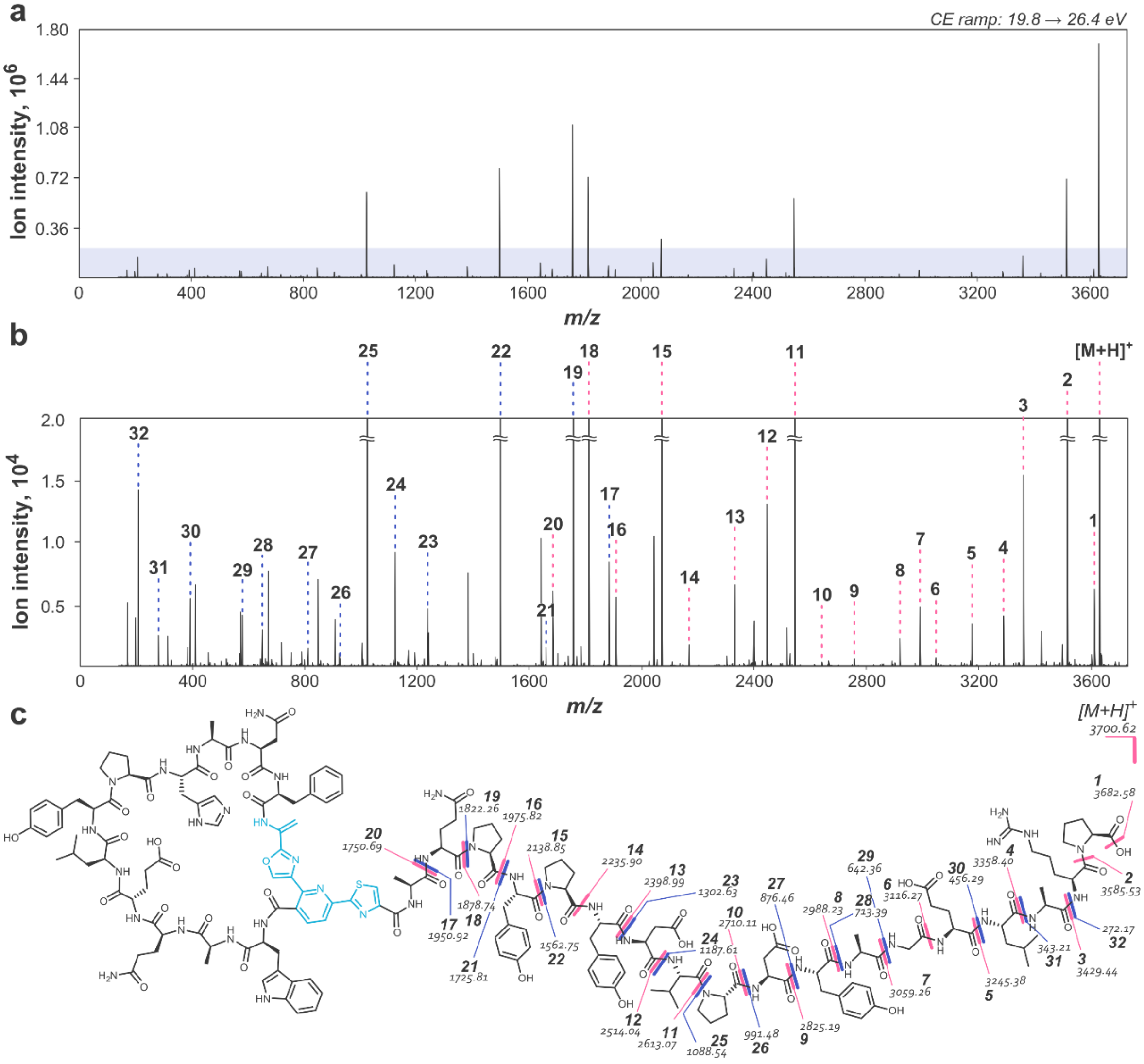

Supplementary Figure 21. Annotated fragmentation spectrum of a 34 amino acid-long pseudo-lactazole synthesized with the FIT-Laz. **(a)** Charge-deconvoluted CID fragmentation spectrum obtained with collision energies ramped from 19.8 to 26.4 eV. **(b)** Y-axis zoom of the shaded area from (a) with spectral assignments; *y*- and *b*-ions are annotated; stable molecule losses (H_2O, NH_3, CO, etc) and double fragmentation assignments are omitted for clarity. **(c)** Assigned chemical structure of the hybrid thiopeptide with mapped assignments. Under these fragmentation conditions, an almost complete *b*/*y*-fragmentation ladder in the tail region was observed, but no double fragmentation (needed to confirm amino acid sequence inside the macrocycle) occurred.

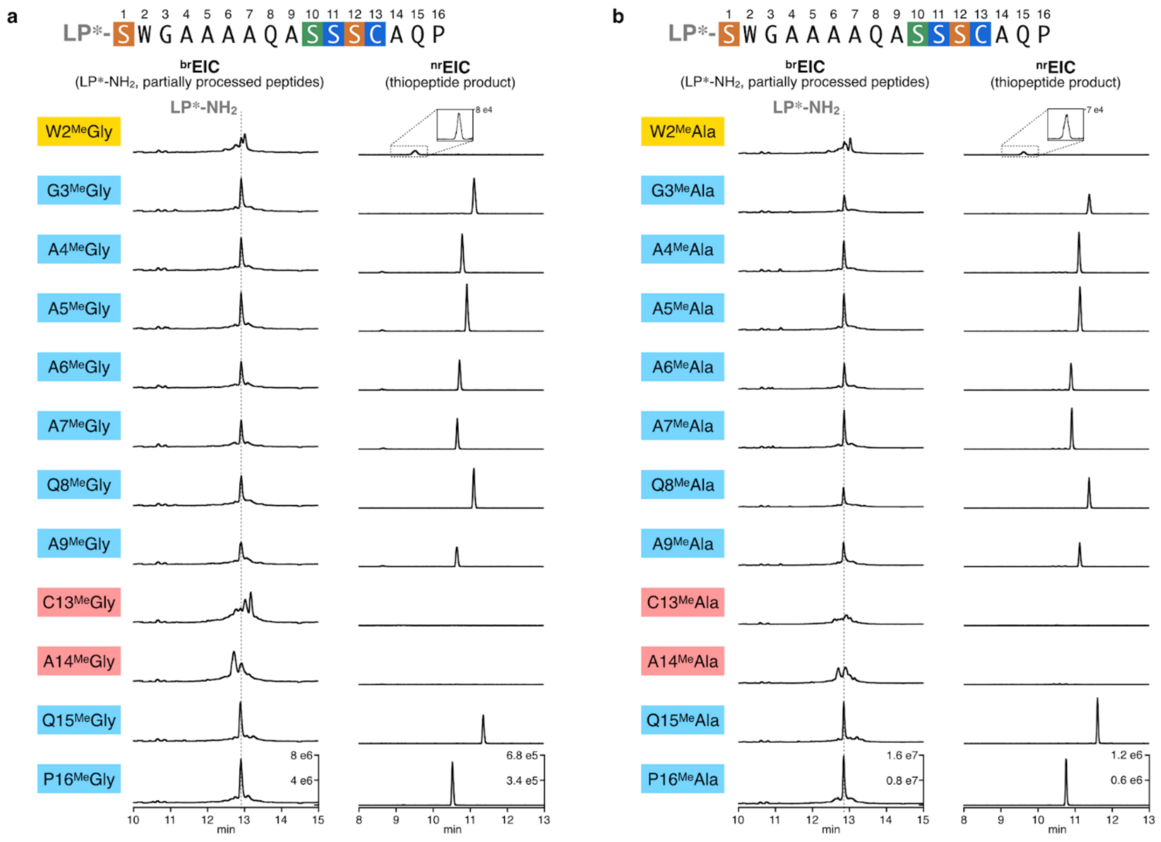

Supplementary Figure 22. Synthesis of *N*-methylated thiopeptides with FIT-Laz. **(a)** ^{Me}Gly-scanning and **(b)** ^{Me}Ala-scanning mutagenesis of LazA^{min}. Precursor peptides accessed with *in vitro* genetic code reprogramming were treated with the full enzyme set and the reaction outcomes were analyzed by LC-MS. Displayed are LC-MS chromatograms (^{br}EIC chromatograms on the left showing partially processed linear peptides and LP*-NH_2 after enzymatic treatment, and ^{nr}EIC chromatograms on the right for expected thiopeptides generated at *m/z* 0.10 tolerance window). LP* stands for LazA LP sequence where formyl-Met is replaced with *N*-biotinylated-Phe (see Supplementary Methods for details).

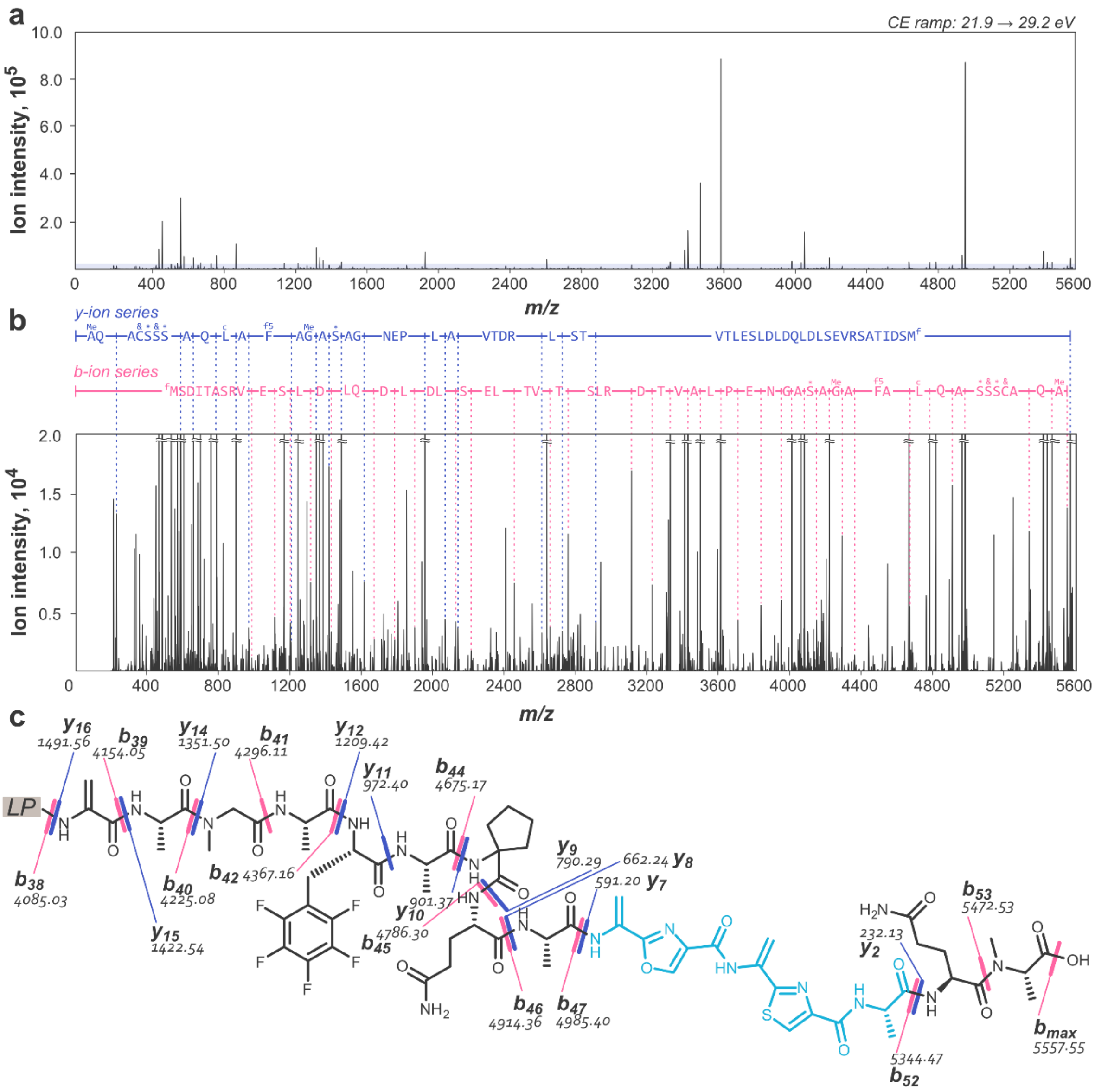

Supplementary Figure 23. MS/MS spectrum of LazA containing 4 npAAs after treatment with LazBDEF/tRNA/GluRS. **(a)** Charge-deconvoluted CID fragmentation spectrum obtained with collision energies ramped from 21.9 to 29.2 eV. **(b)** Y-axis zoom of the shaded area from (a) with spectral assignments; *y*- and *b*-ions are annotated; stable molecule losses (H_2O, NH_3, CO, etc) and double fragmentation assignments are omitted for clarity; "fM" stands for formyl-methionine; asterisks denote dehydration events; ampersands – azole formations. **(c)** Assigned chemical structure of the LazA CP region with mapped annotations; 2 azoles and 2 dehydrations can be localized to the region highlighted in cyan; positions of npAAs (highlighted in red) and the third dehydration match the displayed structure.

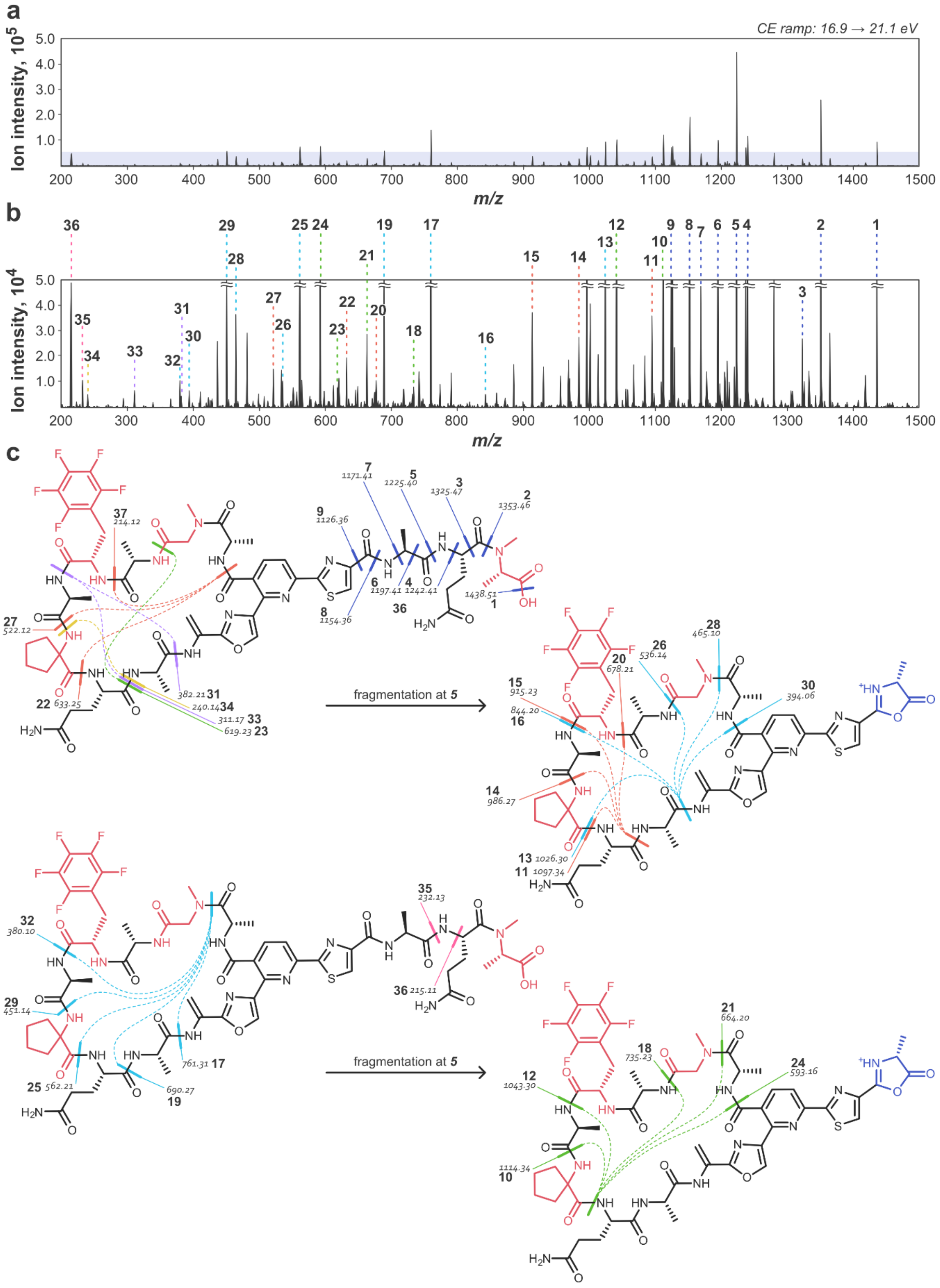
a
CE ramp: 16.9 → 21.1 eV
Ion intensity, 10^5
m/z
b
Ion intensity, 10^4
m/z
c
fragmentation at 5
fragmentation at 5

Supplementary Figure 24. Annotated fragmentation spectrum of the hybrid lactazole thiopeptide containing 4 npAAs. **(a)** Charge-deconvoluted CID fragmentation spectrum obtained with collision energies ramped from 16.9 to 21.1 eV. **(b)** Y-axis zoom of the shaded area from a) with spectral assignments. **(c)** Assigned chemical structure of the hybrid thiopeptide with mapped assignments. Under the acquisition conditions the thiopeptide underwent multiple double fragmentations allowing the mapping of amino acids within the macrocycle. A prominent *b*-type ion 5 underwent further fragmentations, which are displayed separately. Some ions can have multiple potential assignments. In such cases, only 1 isomer is shown. The spectrum enabled unambiguous assignment of positions of npAAs and post-translational modifications.

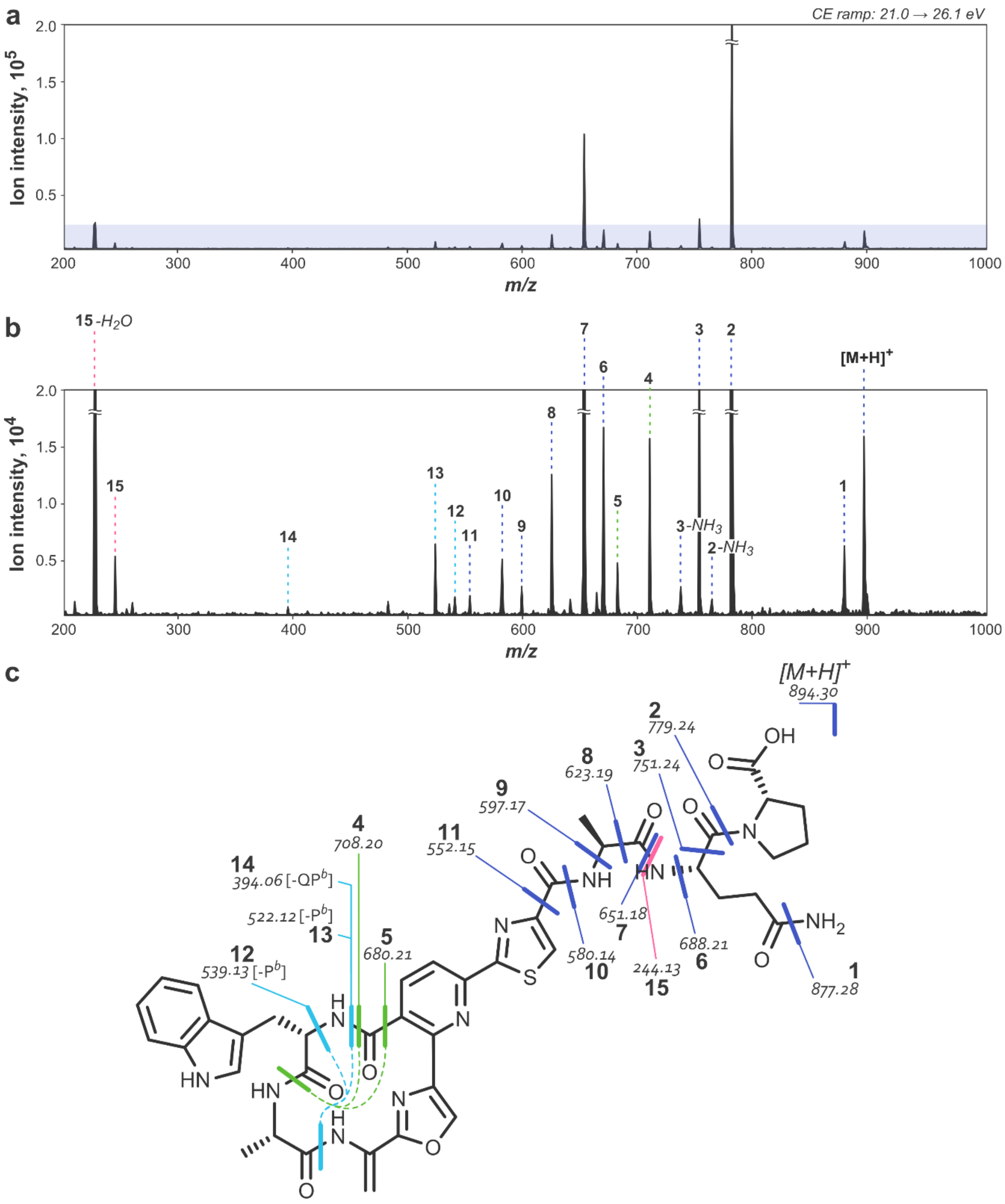

Supplementary Figure 25. Annotated fragmentation spectrum of the 14-membered macrocycle thiopeptide. **(a)** Charge-deconvoluted CID fragmentation spectrum obtained with collision energies ramped from 21.0 to 26.1 eV. **(b)** Y-axis zoom of the shaded area from a) with spectral assignments.

(c) Assigned chemical structure of the thiopeptide with mapped assignments. Under the acquisition conditions the thiopeptide underwent multiple double fragmentations allowing the mapping of amino acids within the macrocycle. A number of triple fragmentations are annotated in cyan; for such assignments, positions of the third fragmentation in the tail region are indicated next to the corresponding *m/z* values. Some ions can have multiple potential assignments. In such cases, only 1 isomer is shown. The majority of observed peaks can be annotated, confirming the assignment.

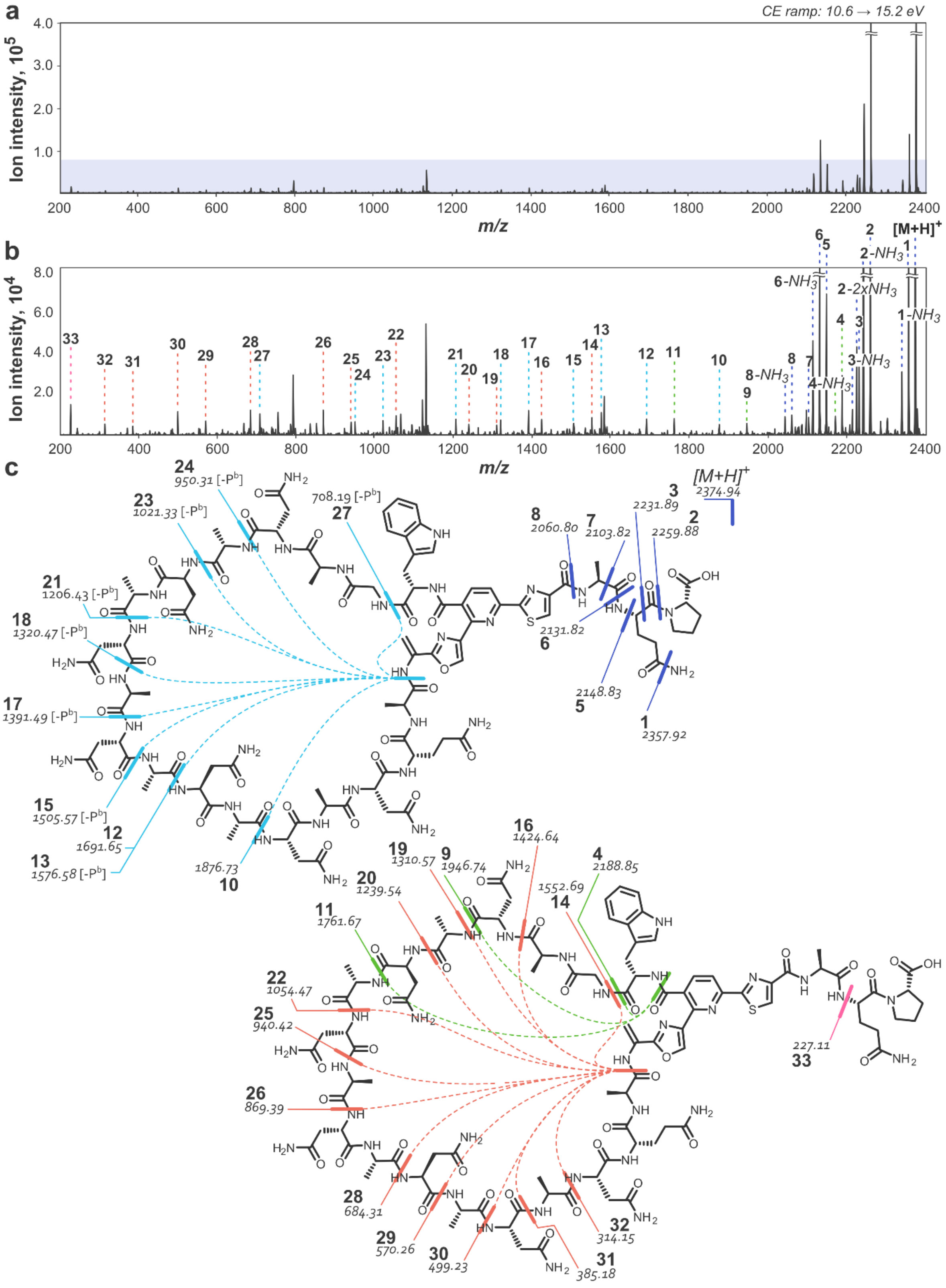

a
CE ramp: 10.6 → 15.2 eV
Ion intensity, 10^5
m/z
b
Ion intensity, 10^4
m/z
[M+H]+
c
[M+H]+
2374.94

Supplementary Figure 26. Annotated fragmentation spectrum of the 62-membered macrocycle thiopeptide. **(a)** Charge-deconvoluted CID fragmentation spectrum obtained with collision energies ramped from 10.6 to 15.2 eV. **(b)** Y-axis zoom of the shaded area from a) with spectral assignments. **(c)** Assigned chemical structure of the thiopeptide with mapped assignments. Under the acquisition conditions the thiopeptide underwent multiple double fragmentations allowing the mapping of amino acids within the macrocycle. A number of triple fragmentations are annotated in cyan; for such assignments, positions of the third fragmentation in the tail region are indicated next to the corresponding *m/z* values. Due to high sequence redundancy, most ions can have multiple potential assignments; only 1 isomer is shown for clarity. Most double fragmentations originating not at the Dha peptide bond are omitted for clarity. Extended double fragmentation ladders inside the macrocycle (cyan and orange) provide strong support to the assigned chemical structure.

Supplementary References

1. Goto, Y., Katoh, T. & Suga, H. Flexizymes for genetic code reprogramming. *Nat. Protoc.* **6**, 779–790 (2011).

2. Shimizu, Y. *et al.* Cell-free translation reconstituted with purified components. *Nat. Biotechnol.* **19**, 751–755 (2001).
