# Supplementary material for: Minimal lactazole scaffold for in vitro thiopeptide bioengineering
Source: Nat Commun. 2020 May 8;11:2272. doi: 10.1038/s41467-020-16145-4 (PMC7210931; doi:10.1038/s41467-020-16145-4)
Supplement: Supplementary file 3 — Description of Additional Supplementary Files [file 41467_2020_16145_MOESM3_ESM.pdf]

Description of Additional Supplementary Files

File Name: Supplementary Data 1
Description: Nucleotide assembly schemes and summary of key LC-MS results. The file contains a list of primers used to assemble DNA templates encoding *lazA* mutants and tRNA/flexizyme templates. Also included are stepwise PCR assembly schemes, aminoacylation conditions, and summary of LC-MS data, including quantification outcomes.

File Name: Supplementary Data 2
Description: A list of ORFs used for heterologous protein expression.
