# Supplementary material for: Minimal lactazole scaffold for in vitro thiopeptide bioengineering
Source: Nat Commun. 2020 May 8;11:2272. doi: 10.1038/s41467-020-16145-4 (PMC7210931; doi:10.1038/s41467-020-16145-4)
Supplement: Supplementary file 5 — Supplementary Data 2 [file 41467_2020_16145_MOESM5_ESM.zip › 242413_1_data_set_4517481_q7zxkh.docx]

## **LazB codon-optimized ORF nucleotide sequence**

CATATGCCGA ATCGCGCCGC ACCCCCTCGT GATGCACGTG CCCCGGTTCC TGCACCGGCC

CCTGCCGCTT ATGCTGCACG TCATGCCCTG GTTCGCAGCA CAGTTCTGGC ATGGCCGGCA

CAGAGCGCAG CCACAGCACA TACCCGTGCC CTGCTGCGCG ATCTGGCAGC CGCAGAAGCA

GCAGCAGAGG CCCTGCGCCC GGCCCTGTGT GATGATCTGT ATGCCGGTCG CGCAGGCCAC

GATGAAGAGT TTCACCGTCG CGTTGTGCTG CCTTTACGCC GCGATCTGCA TAACGGCCGT

ACCCCGCGCG CAGCACTGTT AGACCGCTTA GCAGACCTGC CGCGTCGTAT TCCTCGCCTG

GCCGAATGGC TGGAACTGCG TCGCTTACGC GCACGCCTGC TGGATGCATT AGCCGATGCC

GTTCCGCCTG CACTGGCAGC AGAACGTGCA GCCTTAGCCG ACATTTGCCG TGAACCGGTT

TTCACACGCG CCGTGGCACT GACCAGCGCA GATTTACTGC GCGCAGTTGC CAATACAGCC

GGTGCAACCG GCGAACCGCC TCGCGGCCGT GCCCGTAAAG AAGAAGCAGC CGTTCTGCGC

CACGCACTGC GTGCCACCGC CAAAACCAGC CCTCTGAGCT GGTTCACCGC AGTTGGTTGG

AGTAGCGAGG ATGGCGAAGC AGCAGCCGGT GAACCTCGTG CCTGTGTGCG CGAAAACCGT

GCCCTGGTTA CCGCCCTGGT TCAAGCCCTG CTGGATGACC CTCGTCGCAG TCGTACCCTG

CCGCATCGTA TGACCAGCGC AGCACGCGTG GCAGATGGTC GTGCACGTTA TGCCCGTGCA

GAGGCACTGT TTGCAGGCGG CCGCTTTCTG GTTACCCGCG AAGAAGAAGT GGAACTGGCA

GCCCGCCCTG AGTTAGCACT GCTGGCCAGT TTAGCAGCCA CACCGGCCCC GCCTGACCGT

TTAGCAGCCG GTCTGGCAGA AGCCTTAGGT CGCCCTGGTG GCGATCCTGG CGCACAACGC

TTTGTTGACC AGCTGGTTAC AGCCCGTCTG CTGGTGCCGA CAGAGCCTGT TGATCCTCAG

GATCGTCATC CTCTGCGCAG TCTGGCCGGC TGGTTACGTC AATGGCCGCA AGACGCAGAA

CTGGCACATC GCATTGAGCA GCTGGATCGC CAGAACGCAG AGCTGGCAGT GACAACAGGT

GAACATCGCC CGGAACTGTT AGCCGTGCTG GCCGAGCGTT GGCGTCTGTT ACTGGCCGAT

GCCGGTCGTC CGGTGCCGCA GGAAGCAGCA CCGCTGAGTG TTCTGAGCGA AGACGTTCAC

GCACCGGCCC CTCCGCAACC TCGTCCGGGT GCAGCAGATC GCGCAGCATT AGCCGAACTG

ACAGCCTTAG CCGAGCTGTT TGACCACGCC CATCTGATGC GCCGTGCAGC ACGCGGTCGC

TTTGTGGCAC GCTATGGCGT GGGCGGTGTG TGCGATGCAC CTTGGGATTT TGCCGCCGAC

TTAGCCGACA GTTGGGCAGA TCCGACCCCG CCTGATGAGT TAGCCGCCCT GCGTGAGGAG

TTTGCCAAGC TGCCGGAACA AGATGGTGAG CTGGTTCTGC CGGCAGAACG TATTCGTGCC

CTGGCAGCCC GTTTACCGCA TTGGACAGCC GCACGTCCTT TAAGCTACAG CTGGTTTGTG

CAGCGCGGTA GTGCAGATGG CCTGTTATGC GTGAACCACG TTTACGGCGG TTGGGGTCGC

TTTACCAGCC GTTTTCTGGA CGGTCTGGCA CCTCAGGCCG CAACAGAAGT GGCCCGTCAA

CTGCGCAGTG GTTTAGGTGC CGGTGCACGT GCAGCACAGA TCCGTCCGGT GGGTGGCTTC

AACGCCAACC TGCACCCGCG TCTGTTAGCA GATGAGATTG GTCCTGACCG TCAGTGGACA

GGTCTGGCCG AGAGCGATTT AGACCTGGTT CACGATCCGG TGGACGACCA ACTGCGCCTG

CGCCTGCGTA CAACCGGCGA GCTGCTGGAT GTTCTGTACC TGGGTTTCCT GGCACCGGTG

ATGCTGCCTC GTCGTCTGGG CCCGCTGTTA AATGACCACC CGGAAGGCGT TGTGGATTTC

CGCCCGCTGC TGCCTCGCAC AACCCTGGCC GCACCGGGTG GTACCGTTTT ACGCACACCG

CGCTTACGCC ACCGTCATGT GGTTCTGGCC CGCCGTCGCT GGCTGCTGCC GGCCGGCGTG

TTAGATGCAC TGCGTGCCGA TTTAGCCGCA GATGCAGGTC CTGACGGTGT TCCGGCAGCC

GCAGTGGCAC GTTGGCGCGC ACGTCTGGAC CTGCCTGAAC AACTGTTCTT ACATCCGGCC

CCTGCAGCCG CCGATCCTGC CGGTACCCCG GGCGATGCAT TTGTGGCCCA CCTGCGCGCA

CCGAAACCGC AACCTGTGGA CCTGGGTAAT CCGCTGCACC TGCGCCATCT GGCCAAATGG

CTGACACGCC ATCCTCGTGG CGCCGTTCTG GAAGAGGCAC TGCCGGCAAT CGCCGGTCAT

CCGGAACCTA CCCGCGCCGT GGAACTGGTT GTTGAAACAT ATCGCCCGGG TCGTGGTAGC

GAACAGGCCG CCGGTGCCTT TGAAAGCGTT CGCACCGCAA TTGCAGAAGA AGCCCCTGAC

GAACTCGAG

## **LazC codon-optimized ORF nucleotide sequence**

CATATGAGCG ACCCGGCAGA TGGTCGTGGC GCAGTTACCG CATGGGATGT TGTGCTGTAC

CACTATCGCC CGGATAAAGC CCGTGCACTG CGCGAAGCCG TTCTGCCTCT GGCACGTCAG

GCAGCCGCAG AAGGTCTGGC CGCACACGTG GAACGCCATT GGCGTTTTGG TCCGCATCTG

CGTCTGCGCC TGCGTGGTCC TGAAGCCCGT GTTGCAGGTG CCGCACAGCG TGCAGCAGAA

GCATTACGTG CCTGGGCAGC CGCACATCCG AGTGTTGCCG ATCGCAGTGA CGAACAGCTG

CTGGCAGAGG CCGCAGTTGC AGGTCGCGCA GAGTTAATTG CCCCGCCGTA TGCCCCGCTG

GTGCCGGATA ACACCGTGGT TGCAGCCCCG GCAGATCGTA GCGCAGAAGA TGCCTTACGC

GCCCTGATTG GTGCCGAAAG TGCCGAGCTG CGCGAAGAAC TGCTGCGCAC CGGTCTGCCT

GCCCTGGATA GCGCATGCCA CTTCCTGGGT GCACACGGTG ACACACCGCA GGCCCGTGTG

CAGCTGGTGG TTACCGCACT GGCCGCACAT GCAACCGCAC ACCCTGATGG CCTGGTTGGT

GCCCACTATT CTGTGCTGAG CCATCTGGAA GACTTCCTGG TTCACGAAGA TCCGGATGGT

AGTCTGCGCG CCGCATTTGA ACGCCGTTGG GAACAAAGCG GCCGTGCCGT TACAGCCCTG

GTTGGTCGCA TTGCCGATGG CGGTGCCCGT GATTGGGAAC GTGATTGGGC CCATTGGAGC

GCAACCGCCT GGAGTCTGGC AGAGCGTCGT CTGACAGCCG GTGCAGATCT GGGCGGTCGT

CATGCCGAAT ATCGTGAACG CGCCGAAGCC TTAGGCGATC CGGCCACAGC AGAACGTTGG

AACGCAGAAC TGCGTACCCG CTATAGCGAG TTTCATCGCA TGCTGCAGCG CGCAGACCCG

GATGGTCGTA TGTGGCATCG TCCGGATTAT CTGATTAACC GCGCCGGCAC AAATGGCCTG

TATCGTCTGC TGGCCATCTG CGATGTTCGT CCGATGGAGC GTTATCTGGC AGCCCATCTG

CTGGTGCGCA GTGTTCCTGA GCTGACCGGC CATCGCTGGC AGACCCTGTT AGGCGCCGCA

GAACAGCCGG GTGGTCCTGA ACAGAGTGGT GCAGCAGGCG CAACCGGTGG TGCAGGTCGT

ACCAAACTGG AGGGTGCCGC ACTCGAG

## **LazD codon-optimized ORF nucleotide sequence**

CATATGACCG CAGAACCGGA TGCCGTTCGT CCTCGTTTAC GTCCGGGTGT GGCAGTTACA

CCGCTGCGCG AGGGTCTGCA CTTACGCGGT CGTGAAAGCA GCGTGACATT AGAAGGCAGC

CGTGCACTGC CGGCATTATG GCAGGTTCTG GCAGCCCGTT TAGGCCCGCA GGCAGAGGCA

GCAGATGCAG CCGTGGAAGC CACCGTGGAA CCGCGTGTTG CCGCAGCACT GGCAACCGTG

ACAGCACGCC TGCGTGAGCA CGGCCTGCTG GTTGATCACC CGGATGGCGT GCGCTTACCC

CCTTGGCCGG GCGCAGTTGC AGATGATCCG GGCGGTGCCG AGGCAGCATT AGCAGCAGCC

CGTCCGGTTG TTGCCGCAGC AGATCCGGAT GGCCCTAGTG CCCGCGCAAT GGCACGTGCA

TTAGCACGCG GTGGCACCGC AGCACCGGCA GTTGTGGCAG AACCGGGTTT ACCTGCCGGC

CGTGTGGTTG CCACCGCAGA TGGTCCTGCC GGTACCGAAT TAGCAGTGGC AGTGCAGTGC

GGCGCAGACG GTGGCTTTGT TACCGAACCT GCCGACCCGG CACGTGCACG TACAGACGCA

GCAGCATTAG CAGCACGTCT GGAACCTGCA CCGGTTGCCG ACCCGCCGCC GGTTTTATTA

GCACTGCTGG CCGCCGCAGG TGCACAACGC TTACTGTGCG CAGTGGCCGG TCTGCCTGAT

CCTGGTGAAC CTGCAGATGA CCCGCGCTTA CTGGATGGTC GCCCGACCGT GCTGATTGCA

GATGCAGCAC CGCCGCATGC AGAGCATCAT CCTTGGGCCG CAGGCCCTGG TGCAGTTGCA

GCACCTCCGG GCAGTTTAGC CGAAGCCCTG CGCCGTGTGA ATGCCCTGGG TGATCCGCGC

CTGGGCGTGT TAGACGCACC GAGTGCCGGT GACCTGCCGC AGTTACCGGT GGCACTGGTT

AGCTGCGCCA CACCGGCAGG TCCTTTAGCC GCAGGTGCAG TTCGTACCGA CTTAGCACGC

CTGGCAGCAG CATGCCGTAG TGCCGAACTG CATTTAGCAG CAGTGGGTGG TGGCGCAGTG

CCGGTTGTGG GCGTTGATCC GGATCATGCC TTAGGTCTGG CACTGCGTCG CGCCGTTCTG

GCACGCGCAG TTCGTGGTGA CCGTCCTCTG CCGGATGATC GTGCAGCCCG CGGCGATCGT

ACCGTTCCGG AAAGTGCCTG GCGTGAGCAC CCGCAAGCAG GTCATTGGTA CGGTGTGTTA

GCCCGTCGTC TGGGCCGTGC ACCTGAACCT ACCATTCGTC AGCTGAGCGG CGAGAGCGTG

TACCTGGCCC AGGTTGAAGA AGGTCGTGCC GTTGAAGCAA CCCCGGCAGA TGCCGTTGCC

CATGCAGCAC TGGCAGCACT GACCCGCTTA ATGGCCCGTG GTGCAGGCCT GGCAGCAGTG

CATCATACCG TTTTAAGCGG CGCAGCAGCA CCGTTAGCAG CCGCAGGTCG TACCCCTGCA

GCATGGACCG ACCTGGGCTG GGCAGATCGT TGGCTGGCCG ATATTGCCGA TCGTGAAGCC

GACCTGCACG CCGCACTGGT GCGTATTACC GGTTTACGTA CCGCACGTTG GCGTCCGGCA

ACACCTGAAG CCCGTCCGTT CGCAGATGCC CTGGATGGTT GCGGTTTTAC CGCCCTGACA

GCCGAAGGCG GTCGTCCG

## **LazE codon-optimized ORF nucleotide sequence**

CATATGAGCG AACTGCCGGT TCTGACACCG GTGGAGGCAC TGGCCGCCAC AAGTGGTACA

GCCGTGGTGC ACCTGACCGA ATGGACCCTG GGTCTGGCAG CCCGCTTAAG CCGCCACGCA

TTAGCACATC CGGTGCGCCT GGTTCCGGTT CGTGAAGACG GCGCCTTAAC CGTGGTGGGT

CCTGTTCTGG CACCTGGCGC ACCTGCATGC CTGGCCTGCG TTGAATATCA GCGTCTGGCA

ACCGCAGGTG GTCGCGTGCC GTGGCAGAGT CCTGCCTTAG CCCTGGGTGG TACCGGTACA

CCGGCATTTG CAGAGGCAGT GACAGCCCTG GCAGCCGAAT TAGCCAAGGG TCCGGAGGCA

GCAGAGAGTG CCGAAGGCGC AGGTAGTCCG GAAGCCGCAG AAAGTGCCGA GGGCGCAGGC

AGTCCTGAAG CCGCCGAAAG CGCAGATGGC GCCGGTAGTC CGGAAGCAGC CGAAAGTGCC

GATGGTGCCG GTAGCCCGGA AGCAGCAGAA AGTGCCGACG GTGCAGGTAG TCCGGAGGCA

GCAGAAAGCG CCGATGGCGC AACAGTTCAT GTTGTGCATG GTGGCCGCGC CACATGGAGC

ACCCATCGTG TTCGCCCTGT TGGCGGCTGC GAGGTTTGTC GCCCGTTACC GCCTGATACC

CCTGAGGCAG CCCGCTTACC TGCCACCCCT CGTCCTCTGC CTGATCCGGC AGTTCTGCGC

GGTCCGAATG ACCGTACAGA TGCCGGCCAG CTGCGTGCAG AACTGTACGA TGAGCGTTTT

GGCCCTGTGC GTCGTCTGTT TCGCACAGAA GATAGCGCCT TTGCACTGAC AACCGCATGG

GTGACAGATG GTCGCGCCCT GGATGATGGC GGCTATGGTC GCGCCGCAGA CTTTCGTAGC

AGCGAACGCG TGGCCCTGTT TGAAGCCGTG GAGCGTCATG CAGGTATGCG CCCTCTGGGC

CGTCGCACAG TGTTACGCGC CAGCTATGCA GAACTGGCCC GTGAGCTGGG CCCGGATGCA

GTTTTAGATC CTGCACGTCT GGGTCTGCCG GATGATCCGC ATCAGGGTCA TCCTACACCG

GCAACAGCCC CGTATACACC TGAGCTGGTG TTAGATTGGG TGCACGGTTG GAGTCTGACC

CGCCGTCGTC CGGTTGCAGT TCCGGAACGT GTTGCCTACT GGGAGGTGCC GGGTCGTGAT

CGTCCTCGTG TGGTGTACGA AAGCAGCAAT GGCTGTGGCC TGGGTAATAG CCCTCAAGAA

GCCGCCCTGT ACGGCCTGTT CGAAGTTGCA GAGCGCGATG CCTTTCTGAT GGCCTGGTAT

GCACGCACAC CGCTGCCTGG CGTTGCCGTT CCTACCGAAG ATCCGCAAAT TGCCGAGTTA

GCAGACCGCG CCGAGTTATT CGGTTATCAC CTGACCCTGC TGGATGCAAC CAACGATCTG

GGTGTGCCGG CCGTGATCGC CCTGTGTCGT CATCGTGGCG ACCATCCGGA CGCACCTCGC

ACATTACTGG CCGCAGGTGC CCACCATGAT CCGCGCACAG CCATTCGTAG CGCCGTTGCC

GAAGTGGTGA CAAATGTTCA AGAAGCTCCT CATCGTAGCA CAGCACCGGG TGGTCCGCGT

GACCCTCAGC GTTTACGTCC GATGCTGGAG CGTCCGGAAC TGGTGGTGAG CCTGGACGAT

CATGTGGGTC TGAACACCCT GCCGGAAGCA CAGCCTCGTC TGGACTTTCT GTTTGCCGGC

CCCCCTCCGG TGCCTTGGAC AGAACGCTGG CCTGGCGATC CGGAACCGGT GACCGATCTG

ACAGATCTGT TAGAGCGCAC AGTGACACGC CTGGCAGGTG AAGATCTGGA GGTGCTGGTT

GTTACCCAGG ATGAGCCGGG CGTTCGTGAC CGCTTAGGTC TGCATTGCGC CAAAGTTGTT

GTGCCGGGTA CACTGCCTAT GACCTTCGGC CATGCAAATC GTCGCACACG CGGCTTAAGC

CGTCTGCTGG AGGTGCCGTA TCGCTTAGGT CGTACACCGG CCCCTTTACG CCACGATGAA

TTACCGCTGC ATCCGCATCC TTTTCCT

## **LazF ORF nucleotide sequence**

ATGACCA CCCACGCGCT GCCGGCCACC ACCTGGCACA

GCCTGCACCT CGCGCTGCCG CTGCCCGCCC GCGAGGCCGA CGCCTTCCTC ACCGAGGACC

TCGCCCCGCT GATGGACGGG CTCGCCGGCA CCGACTGGTT CTTCATCCGC TACGGCGAGG

GCGGCCCCCA CCTGCGCATC CGCCACCGCG GCCCGGGCCC GGCGCCCGCC TCCCTCGCCG

CCGACCTCAC CCGCCTCGCC ACCCGACGCA CCGCGCCCGA CGGCCCGTTC GCGGACGGGC

ACGGCACCGT GACGGAGGTC CCGTACGAGC CCGAGACCGA GCGCTACGGC GGCGCGGCCC

TGCTGCCGAT CGCCGAAGAG GTGTTCACCC ACTCCACCCG CGCCGCCGTC CGCGCCCTGC

ACGCCCTCGG CGCGGCCCCC GAGAAGCGGT TGCAGCTCGC CCTCGACCTC GCCCACACCA

CCGCGTACGC GCTCGGCCTC GACGAACTCG CCGCCTCCCG CTGGCTGCGC CGCCACGCCG

CCGCCTGGCG CTGGGTCACC GAGTTCCGGC CGCTGCCGGG CGCCGCCGTG CACACCCGGG

TCAACACCGT GTTCGCCCGC CAGCGGGAGA CGCTGGCCCG CCGCGCCCGG GAGCTGCGCG

CGGCACTGGA CGCCGGCACG GCCAGCCCCT GGCTGGGGGA CTGGGCGGCG CGGGCCGCCG

AGGCCGCCGC CCGGATGCGG GCCGTCGCGG CCGCCGAGGC CGCCGCGCCC GCCTCGGCCG

CCGAGGAGGC CGAGGAGCGC CTGGAGTGGA TCTGGGCCTC CCAGCTGCAC ATGCTGTTCA

ACCGGCTCGG CGTCGGCCCC GACGAGGAAC GGGCGGTCTG CCGCCTCGCC GCCCGCACCC

TGCTGGAGAC CGGACAGCCG TTCACCTTCT TCCCCGCCGA CCACCACGCC CCCGACCACC

AGTACCTGGA ACGCAGCAAG TTCCAGATCG GCCGCGGCGA GGACACCGCC CTGCGCGACC

TGCCGCACGA CCCGGCCCCG AGCCCCCGCC CCGGCGACCT CCCGCTGCCC GCCGACCCGC

TGCCGCCCGT CACCCTCGCC GACGCCCTGC GCACCCGCCG CTCCACCCGC GGCCCGCTGA

CCGGCCCGCT CACCGCCGGC GCGCTCGGCG GACTGCTCTG GTCCGCCTTC GCCCCCGCCC

CCGACACCGG CCACCGCCCG TACCCCAGCG CGGGCGCCCT GCACACCGTC CGGCTGCGCC

TGCTCGCCCT CGCCGTCGAC GGCCTGCCCG CCGGCACCTA CCACTGCCTC CCCGAACACC

GCAGCCTGCG CCCGATCGGC CCGGCCCCCG CCCTCGACGA CCTCAAGGCG CTCTCCTCCT

ACCTCTCCCG CCCGGCCGAG GACCCCGACG CCATCGGCGT CGACCGGGCC CCCGCCGTCC

TCGCCGTGTA CCTCGACCTC GCCCGGCTGC GCCGCCGCTA CGGTCTGCGC GCCCTGCGCC

TCGGCGTCCT GGAAGCCGGA CACCTGGCCC AGAACCTGCT CCTGACCTCC GCCGCCTTCG

GCCTCGGCAC CACCCCCCTC GGCGGCCTCC AGGACGACCT CGCCCACGAA CTCCTCGGCC

TGGACGACCT CGGCGAGCCG ATCCAGTACC TGCTCCCGCT CGGCCGGCCG GGGACTGTAC

CGGTGATCAT GGAGTGA

## ***S. lividans* GluRS codon-optimized ORF nucleotide sequence**

GTGGCTAGCG CATCCGGCTC CCCCGTACGC

GTCCGTTTCT GTCCGTCCCC CACCGGCAAC CCCCACGTGG GCCTGGTCCG CACCGCCCTG

TTCAACTGGG CCTTCGCGCG CCACCACCAG GGCACCCTGG TCTTCCGCAT CGAGGACACC

GACGCCGCCC GCGACTCCGA GGAGTCGTAC GACCAGCTGC TCGACTCGAT GCGCTGGCTG

GGCTTCGACT GGGACGAGGG TCCCGAGGTC GGCGGCCCGC ACGCGCCGTA CCGCCAGTCG

CAGCGCATGG ACATCTACCA GGACGTCGCC CAGAAGCTCC TGGACGCCGG CCACGCCTAC

CGCTGCTACT GCTCCCAGGA GGAGCTGGAC ACCCGCCGCG AGGCCGCCCG CGCCGCCGGG

AAGCCCTCCG GCTACGACGG CCACTGCCGC GAGCTGACCG ACGCACAGGT CGAGGAGTAC

ACGTCCCAGG GCCGCGAGCC CATCGTCCGC TTCCGGATGC CCGACGAGGC GATCACCTTC

ACGGACCTGG TCCGCGGCGA GATCACCTAC CTGCCGGAGA ACGTCCCGGA CTACGGCATC

GTCCGCGCCA ACGGGGCGCC CCTCTACACG CTGGTCAACC CCGTCGACGA CGCGCTGATG

GAGATCACCC ACGTCCTGCG CGGCGAGGAC CTGCTCTCCT CCACCCCGCG CCAGATCGCC

CTGTACAAGG CGCTGATCGA GCTGGGCGTC GCCAAGGAGA TCCCCGCCTT CGGCCACCTG

CCGTACGTCA TGGGCGAGGG CAACAAGAAG CTCTCCAAGC GCGACCCGCA GTCGAGCCTC

AACCTCTACC GCGAGCGCGG CTTCCTCCCC GAGGGCCTGC TCAACTACCT CTCCCTCCTC

GGCTGGTCGC TCTCGGCCGA CCAGGACATC TTCACGATCG AGGAGATGGT CGCGGCCTTC

GACGTCTCCG ACGTCCAGCC CAACCCGGCC CGCTTCGACC TCAAGAAGTG CGAGGCGATC

AACGGCGACC ACATCCGCCT GCTGGAGGTC AAGGACTTCA CCGAGCGCTG CCGCCCCTGG

CTGAAGGCCC CCGTCGCCCC CTGGGCGCCG GAGGACTTCG ACGAGGCCAA GTGGCAGGCG

ATCGCGCCGC ACGCGCAGAC CCGCCTGAAG GTCCTCTCCG AGATCACCGA CAACGTCGAC

TTCCTGTTCC TGCCGGAGCC GGTCTTCGAC GAGGCCAGCT GGACCAAGGC CATGAAGGAG

GGCTCGGACG CGCTCCTGAC CACGGCCCGC GAGAAGCTGG ACGCCGCCGA CTGGACCTCC

CCGGAGGCCC TCAAGGAGGC CGTCCTGGCC GCCGGTGAGG CCCACGGTCT CAAGCTCGGC

AAGGCCCAGG CCCCCGTCCG CGTCGCCGTC ACCGGCCGCA CGGTCGGCCT GCCCCTCTTC

GAGTCCCTGG AGGTCCTGGG CAAGGAGAAG GCACTGGCGC GCATCGACGC GGCGCTGGCG

CGACTGGCGG CGTAA
