# Supplementary material for: Minimal lactazole scaffold for in vitro thiopeptide bioengineering
Source: Nat Commun. 2020 May 8;11:2272. doi: 10.1038/s41467-020-16145-4 (PMC7210931; doi:10.1038/s41467-020-16145-4)
Supplement: Supplementary file 6 — Reporting Summary [file 41467_2020_16145_MOESM6_ESM.pdf]

nature research

Corresponding author(s): Hiroaki Suga
Hiroyasu Onaka

Last updated by author(s): Mar 30, 2020

Reporting Summary

Nature Research wishes to improve the reproducibility of the work that we publish. This form provides structure for consistency and transparency in reporting. For further information on Nature Research policies, see Authors & Referees and the Editorial Policy Checklist .

Statistics

For all statistical analyses, confirm that the following items are present in the figure legend, table legend, main text, or Methods section.

n/a	Confirmed	
	x	The exact sample size (*n*) for each experimental group/condition, given as a discrete number and unit of measurement
	x	A statement on whether measurements were taken from distinct samples or whether the same sample was measured repeatedly
x		The statistical test(s) used AND whether they are one- or two-sided<br>*Only common tests should be described solely by name; describe more complex techniques in the Methods section.*
x		A description of all covariates tested
x		A description of any assumptions or corrections, such as tests of normality and adjustment for multiple comparisons
	x	A full description of the statistical parameters including central tendency (e.g. means) or other basic estimates (e.g. regression coefficient) AND variation (e.g. standard deviation) or associated estimates of uncertainty (e.g. confidence intervals)
x		For null hypothesis testing, the test statistic (e.g. *F*, *t*, *r*) with confidence intervals, effect sizes, degrees of freedom and *P* value noted<br>*Give P values as exact values whenever suitable.*
x		For Bayesian analysis, information on the choice of priors and Markov chain Monte Carlo settings
x		For hierarchical and complex designs, identification of the appropriate level for tests and full reporting of outcomes
x		Estimates of effect sizes (e.g. Cohen's *d*, Pearson's *r*), indicating how they were calculated

Our web collection on statistics for biologists contains articles on many of the points above.

Software and code

Policy information about availability of computer code

Data collection	LCMS data was acquired on a Waters Xevo G2-XS QTof instrument under control of MassLynx v.4.1 as defined in Methods and Supplementary Methods.
Data analysis	LCMS data and MS/MS fragmentations spectra were analyzed in MassLynx v.4.1 and Unifi v.1.8.2.169, respectively. Details of data analysis are summarized in Methods. Expasy ProtParam (https://web.expasy.org/protparam/) and RandSeq (https://web.expasy.org/randseq/) tools were used for protein analysis and generation of randomized sequences, respectively. No custom written code was used.

For manuscripts utilizing custom algorithms or software that are central to the research but not yet described in published literature, software must be made available to editors/reviewers. We strongly encourage code deposition in a community repository (e.g. GitHub). See the Nature Research guidelines for submitting code & software for further information.

Data

Policy information about availability of data

All manuscripts must include a data availability statement. This statement should provide the following information, where applicable:

- Accession codes, unique identifiers, or web links for publicly available datasets
- A list of figures that have associated raw data
- A description of any restrictions on data availability

The manuscript included a data availability statement. It reads: "Primer sequences, codon optimized ORFs used for protein expression, DNA template assembly schemes, and summary of LC-MS data are available in Supplementary Data. Other results are available upon reasonable request from the corresponding authors."

Field-specific reporting

Please select the one below that is the best fit for your research. If you are not sure, read the appropriate sections before making your selection.

☒ Life sciences ☐ Behavioural & social sciences ☐ Ecological, evolutionary & environmental sciences

For a reference copy of the document with all sections, see nature.com/documents/nr-reporting-summary-flat.pdf

Life sciences study design

All studies must disclose on these points even when the disclosure is negative.

Sample size	For thiopeptide quantification, n=3 independent samples were chosen to test the reproducibility of the experiment.
Data exclusions	No data was excluded.
Replication	Syntheses of lactazole A and minimal lactazole scaffold were repeated at least 10 times each. Syntheses of the 34 amino acid-long thiopeptide, the thiopeptide with 4 npAAs, the 14-membered macrocycle and the 62-membered macrocycle thiopeptides were repeated three times each. All in vitro reconstitution experiments were performed 3 times, and ring contraction/expansion study was done twice. Each protein was purified at least twice during the course of the study. All attempted experiments were reproducible; LCMS chromatograms shown in the manuscript are representative of an average trial.
Randomization	Sequence randomization for experiments in Figure 4 was performed as described in Supplementary Methods
Blinding	Experimental designs did not require and were incompatible with blinding. No experiments requiring blinding were performed.

Reporting for specific materials, systems and methods

We require information from authors about some types of materials, experimental systems and methods used in many studies. Here, indicate whether each material, system or method listed is relevant to your study. If you are not sure if a list item applies to your research, read the appropriate section before selecting a response.

Materials & experimental systems

n/a	Involved in the study	
☒	☐	Antibodies
☒	☐	Eukaryotic cell lines
☒	☐	Palaeontology
☒	☐	Animals and other organisms
☒	☐	Human research participants
☒	☐	Clinical data

Methods

n/a	Involved in the study	
☒	☐	ChIP-seq
☒	☐	Flow cytometry
☒	☐	MRI-based neuroimaging
